# Influence of Plant Species and De‐Icing Salt on Microbial Communities in Bioretention

**DOI:** 10.1111/1758-2229.70193

**Published:** 2025-09-07

**Authors:** Henry Beral, Jacques Brisson, Margit Kõiv‐Vainik, Joan Laur, Danielle Dagenais

**Affiliations:** ^1^ Institut de recherche en biologie végétale, Département de sciences biologiques Université de Montréal Montreal Québec Canada; ^2^ Institute of Ecology and Earth Sciences University of Tartu Tartu Estonia; ^3^ École d'urbanisme et d'architecture de paysage, Faculté de l'aménagement Université de Montréal Montréal Québec Canada

**Keywords:** biofilter, cold climate, dormancy, green infrastructure, microbial communities, rain garden, salinity

## Abstract

Bioretention (BR) systems are green infrastructures used to manage runoff even in cold climates. Bacteria and fungi play a role in BR's performance. This mesocosm study investigated the influence of plant species and de‐icing salt on the diversity, the community composition, and the differential abundance of bacteria and fungi in BR. *Cornus sericea, Juncus effusus, Iris versicolor* and *Sesleria autumnalis* were selected. They are planted in BR while differing in terms of biological forms and functional traits. The semi‐synthetic stormwater used was supplemented in spring with four NaCl concentrations (0, 250, 1000 or 4000 mg Cl.L^−1^). Soil was sampled before the experiment, before salt application, and 5 months after the end of the salt treatment. The bacterial and fungal taxa were characterised by sequencing the 16S and ITS regions. The bacteria and fungi found in the BR were adapted to a cold, humid, and contaminated environment. No differences in microbial communities and their functions between treatments were perceivable 5 months after salt treatment. The taxa abundantly present are involved in functions related to the nitrogen cycle, degradation of hydrocarbons, metals tolerance, and remediation. Some were putative plant beneficial symbionts. The presence of certain microbial taxa varied significantly between plant species.

## Graphical abstract

Plant species shape bioretention bacterial and fungal communities and their associated functions, which drive system efficiency. These results underscore the importance of plant selection when designing bioretention systems. Moreover, the communities were preserved or recovered within 5 months after the exposure to NaCl levels typical of spring runoff. These findings are promising for the use of bioretention systems in cold climates where de‐icing salts are commonly applied.

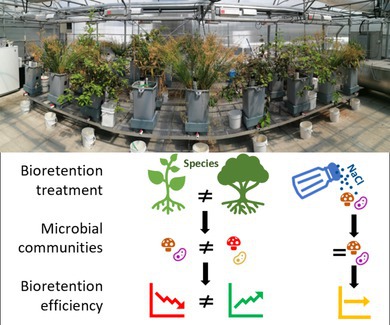

## 1 Introduction

Bioretention cells (BRs) also known as rain gardens or biofilters, are green stormwater infrastructures designed to mitigate the hydrological and pollutant impacts of urban runoff. By reducing water volume, delaying peak flows, and removing pollutants such as nutrients, trace metals, and hydrocarbons, BRs contribute to more sustainable urban water management (Kratky et al. 2017). Their effectiveness is largely influenced by interactions among physical, chemical, and biological components. While substrate composition and vegetation have been extensively studied as critical design features (Nazarpour et al. 2023), the contributions of bacterial or fungal communities remain relatively underexplored, particularly in cold‐climate contexts where de‐icing salts introduce seasonal stress (Skorobogatov et al. 2020).

Microorganisms in BR have the potential to enhance nutrient cycling, degrade hydrocarbons, immobilise metals, and support plant health and growth, similar to roles observed in other green infrastructures such as treatment wetlands (Faulwetter et al. 2009; Marchand et al. 2010; Wang et al. 2016, 2017), bioswales (Brodsky et al. 2019) and green roofs (Hénault et al. 2022). These microbial functions are increasingly recognised as relevant to BR systems. The structure and function of microbial communities are dynamic and can be shaped by system design factors and management practices.

Among the multiple environmental variables that affect microbial dynamics in BRs, previous studies have shown that media texture and composition, the presence of saturated zones, and the chemistry of synthetic runoff have an influence (Fraser et al. 2018; Zuo et al. 2020; Fan et al. 2022). However, two additional variables remain poorly studied: plant species identity and exposure to de‐icing salt. Vegetation may act as a biotic filter that selects specific microbial taxa based on root traits such as exudate profiles and rhizosphere oxygenation.

The use of de‐icing salts, primarily sodium chloride (NaCl), introduces episodic abiotic stressors into BR systems. Runoff resulting from salt applications can deliver pulses of chloride, which may alter soil chemistry, induce osmotic stress, cause pH shifts, and create nutrient imbalances. These changes can compromise microbial viability and impair essential functions (Canada Environment 2011; Denich et al. 2013). Despite their frequent co‐occurrence, interactions between plant species and salt stress are rarely studied in tandem. Little is known about how plant species may buffer or exacerbate the effects of salt on microbial communities. Root traits such as exudate chemistry, salt exclusion, and mycorrhizal associations could potentially enhance microbial resilience (Poor et al. 2018; Mehmood et al. 2021). Conversely, high salt concentrations may override plant‐driven microbial recruitment, potentially leading to reduced microbial diversity or impaired system performance.

Understanding how microbial communities respond to plant identity and salt exposure is crucial not only for ecological insight but also for optimising BR design, particularly in regions with heavy winter salt applications. For example, plant species capable of recruiting salt‐tolerant or functionally diverse microbial taxa could improve the long‐term stability of microbial services such as nitrification, denitrification, hydrocarbon degradation, and metal tolerance. Bacterial taxa such as *Nitrosomonas*, *Nitrospira*, and *Pseudomonas*, and fungal groups such as *Serendipita* or *Trichoderma*, are examples of organisms with important roles in nitrogen cycling and plant symbiosis that may be differentially affected by vegetation or salt (Davis et al. 2010; Palacios and Winfrey 2021).

Moreover, while bacterial communities have received more attention in BR research, fungal communities may be equally important. Mycorrhizal fungi can enhance plant nutrient uptake and stress tolerance, while saprotrophic fungi contribute to organic matter turnover and phosphorus cycling (Taylor et al. 2018). The extent to which fungi respond to plant species and salinity in BR systems has not yet been documented, and few studies have simultaneously examined bacterial and fungal dynamics across vegetation types under environmentally realistic salt exposures. This is a notable gap given fungi's potential to complement bacterial processes and increase BR system resilience under fluctuating environmental conditions.

To address these knowledge gaps, we conducted a year‐long BR mesocosm experiment simulating seasonal conditions in cold‐climate. Four plant species with distinct morphological and functional traits were selected based on their common use in BR systems. Simulated stormwater runoff containing varying concentrations of NaCl was applied during spring to mimic typical salt‐laden runoff. Soil samples were collected at three time points and analysed using high‐throughput sequencing of bacterial (16S rRNA) and fungal (ITS) regions, followed by functional prediction to assess ecological roles.

The specific objectives of this study were to:

1. Characterise baseline bacterial and fungal communities across different plant species under temperate seasonal conditions.
2. Evaluate the extent to which plant identity shapes microbial diversity and community structure.
3. Determine whether short‐term de‐icing salt exposure induces lasting microbial shifts.
4. Assess whether vegetation can mediate or buffer a possible impact of de‐icing salt on microbial communities.

By integrating vegetation and realistic de‐icing salt exposure into a unified experimental framework, this research contributes to the ecological understanding of BR microbial communities. Ultimately, these insights can guide plant selection and management practices in BRs, supporting the design of more resilient and functionally robust systems in cold urban climates.

## 2 Experimental Procedures

**FIGURE 1:**
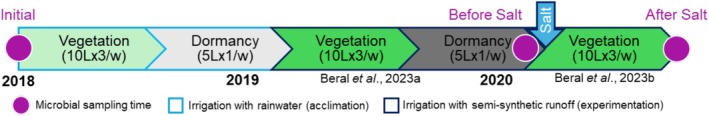
Experimental timeline including watering phases (Vegetation phases in green: 10 L three time a week; Dormancy phases in grey: 5 L once a week) and microbial sampling times (purple circle; initial: 01/06/2018; before salt: 28/02/2020; after salt: 25/08/2020).

The study was conducted from June 2019 to August 2020 in an experimental BR mesocosm setup that was operated since spring 2018 in the ‘Phytozone’ greenhouse of the Institut de recherche en biologie végétale (IRBV) on the research site of the Montreal botanical garden, in Canada (Figures 1 and S1). At the start of the experiment, the plants were thus well established, the BR mesocosms having already gone through one full year of maturation under a continuously simulated summer‐winter regime of rainwater or simulated runoff (Beral et al. 2023a, 2023b). The experimental design included three key sampling timepoints: prior to planting, after 1 year of plant establishment but before salt application, and 5 months following salt exposure. These timepoints were selected to directly correspond to our research objectives. Specifically, the second sampling (pre‐salt) enabled us to isolate and assess the influence of plant species and BR conditions (simulated stormwater, water regime, substrate) on microbial communities, while the last sampling (post‐salt) allowed us to evaluate both the residual effects of salt and the potential buffering role of vegetation.

### 2.1 Mesocosm Set‐Up

Twenty‐five mesocosms (dimensions: H78 × L35 × W35 cm) were constructed using reinforced containers made of high‐density polyethylene and polypropylene plastic. Each mesocosm was equipped with a perforated drainage pipe in the bottom layer (diam. 1.9 cm) connected to a vertical aeration pipe of the same diameter. In the bottom, a 10 cm layer of granite gravel (Ø 5–12 mm; ‘Granite gris du nord’ from Agrebec Inc.) was placed for drainage (Figure S1). The main media consisted of a 60 cm layer of a commercial sandy loam BR media (‘Natureausol’ from Savaria Inc.; characteristics summarised in Table S1). On top of the main media, 3 cm of Savaria Inc. ramial wood chips were added as a mulch layer. The mesocosms were planted with either *Juncus effusus, Cornus sericea, Sesleria autumnalis* or *Iris versicolor* (from now on, called: *Cornus, Juncus, Sesleria* or *Iris*), or left unplanted, with 5 replicates for each option. More information on the experimental design can be found in Beral et al. (2023a, 2023b). These species were selected because commonly used in BR due to their ornamental value and their tolerance to such system, and because they were included in the Canadian Standards for BR design in use at the time of the study (Standards Council of Canada 2019).

Nowadays, it is a common practice for municipalities to add mycorrhizae when planting. This technique can improve plant establishment and growth (Maronek et al. 1981; Azcón‐Aguilar and Barea 1997). Thus, at the time of planting, the mycorrhizal inoculant ‘MykePro Landscape’ from Premier Tech Ltd. (containing *Glomus intraradices, Pisolithus tinctorius*, *Scleroderma cepa*, *Scleroderma citrinni*, *Rhizopogon roseolus*, *Rhizopogon subscaerelescens*, *Rhizopogon villosulus*, *Rhizopogon vulgaris*, *Laccaria laccata*) was added to the mesocosm according to the manufacturer's recommendations (30 mL/plant in the planting hole applied).

### 2.2 Simulated Urban Runoff

The urban runoff application volume and frequency was based on regional climatic data for rainfall (Environment and Climate Change Canada 2018) and on the recommended drainage area for BR of 10% (as recommended by Coffman et al. 1993; Yang and Chui 2018; the Standard Council of Canada, 2019). As a result, each mesocosm was irrigated with 10 L, 3 times per week (Monday, Wednesday and Friday) during the growing period (6 months; March–September) and with 5 L, once a week (Monday) during the dormant period (October–February; Figure 1). During the acclimation period of 12 months (from June 2018 to June 2019), the mesocosms were irrigated simply with rainwater collected from the roof of the greenhouse (average electrical conductivity 20 ± 3 μS.cm^−1^). Then, the experiment was conducted in two phases. During the first phase, from June 2019 to late February 2020, the mesocosms were irrigated with semi‐synthetic runoff consisting of macro‐ and micro‐nutrients, metals, and a carbon source (Table S2). This phase allowed us to evaluate the effect of plant presence and of species on BR performance. More details on the plant species experiment can be found in Beral et al. (2023b). For the second phase (from March 2020 until August 2020), the irrigation followed the same simulated runoff application as previously, but for a short period of time (from March 12 to March 23), the runoff was supplemented during four consecutive times with de‐icing salt to mimic a spring‐time salty runoff. Four concentrations were tested on one individual of each plant species and unplanted control (Figure S1A). The tested NaCl concentrations were (1) 0 mg Cl.L^−1^ as negative control, (2) 250 mg Cl.L^−1^ (~0.8 mS/cm) corresponding to the maximum Cl concentration recommended by the Canadian drinking water guidelines (Health Canada 1987, 1997), (3) 1000 mg Cl.L^−1^ (~3.3 mS/cm) concentration usually found in runoff from roads spread with a mixed 95/5 sand/salt mixture and already tested in previous scientific studies, or (4) 4000 mg Cl.L^−1^ (~13.2 mS/cm) concentration usually found in runoff from roads spread with pure salt (NaCl) (Mayer et al. 1999; Denich et al. 2013; Paus et al. 2014; Géhéniau et al. 2015; Taka et al. 2017). Since two *Sesleria* replicates died during the first phase of the experiment, this species was not retained for the second phase. Also, in order to respect a Latin square configuration for statistical purposes, only four replicates for each of the three species + unplanted controls were kept for the salt experiment (unplanted and planted with *Iris*, *Juncus* and *Cornus*) for a total of 16 mesocosms. More detail on the de‐icing salt experiment can be found in (Beral et al. 2023a).

### 2.3 Sampling

Three sampling campaigns were carried out to match our experimental objectives: (1) an initial sampling of the nursery substrate before planting, (2) a pre‐salt sampling after 1 year of vegetation establishment and simulated runoff exposure, with the aim to capture plant species identity and BR conditions on microbial communities, and (3) a post‐salt sampling 5 months after the end of a spring salt pulse, intended to detect lasting impacts of de‐icing salt and any interactions with plant species (summarise in Figure 1). This delay was intentional in order to avoid destructive sampling during the operational phase, which could have compromised system integrity and performance monitoring (hydraulics in particular). Sampling at this later stage still allowed us to assess whether any changes in microbial communities persisted over time or if recovery had occurred after salt exposure. Finally, this design enabled a clear separation between vegetation and salt effects in subsequent statistical analyses. In June 2018, we collected initial samples of the nursery substrate directly from the pots in which the plants were grown, with three replicates per plant species. These samples included both the bulk substrate and roots, which were carefully included in the sampling to capture the root‐associated microbial and soil community present prior to transplanting into the mesocosms. Three samples from the mesocosms substrate, representative of Unplanted, were also collected, for a total of 15 samples. At the end of the first phase, just before salt runoffs (February 28, 2020), we took cores from the 5 replicates of the unplanted and the 4 plant species, for a total of 25 samples. Then, 5 months after the end of the spring salt runoff tests (August 25, 2020), we took cores from the unplanted mesocosms and the 3 remaining plant species tested with the 4 NaCl concentrations, for a total of 16 samples. The cores were taken horizontally at −10 cm from the surface of the main substrate. After the columns were pierced with a hole saw, coring was done with a brass pipe (diameter 3.8 cm and length 15 cm). The pipe was washed with bleach and rinsed 3 times before each sampling. All cores were immediately stored at −20°C after collection, until the DNA extraction day. On this day, cores were thawed and homogenised prior to sampling. The 0.25 g soil samples included rhizosphere except for unplanted mesocosms. This sampling allowed us to test the combined effect of plant species, runoff and salty runoff application on bacterial and fungal communities.

### 2.4 DNA Extraction, Purification and Sequencing

The DNA extraction and purification were performed in the beginning of 2021 using DNeasy PowerSoil Pro Kit according to the manufacturer's protocol (QIAGEN, Hilden, Germany). Then, DNA were repurified following Ethanol precipitation of nucleic acids protocol (OpenWetWare contributors 2012), except for the resuspending done in 50 μL of TE (1 M Tris–HCl, pH 7.5, 1 mM EDTA). Prior to sequencing, GenomeQc inc. has prepared the library after the target enrichment via Polymerase Chain Reaction (PCR) with 16S ribosomal RNA gene (16S rRNA) primers for the bacteria and internal transcribed spacer (ITS) primers for fungi described in ‘Earth Microbiome Project’ (2022) as summarised in Table S3. These primers were selected as they have a large phylogenetic coverage (Baker et al. 2003; Throback et al. 2004; Hornek et al. 2006; Chen et al. 2013, 2016, 2019; Pester et al. 2014; Ramanathan et al. 2017; Keeley et al. 2020; Tilstra 2020; Wang and He 2020).

### 2.5 Bioinformatics

Paired‐end sequences obtained by Illumina‐sequencing MiSeq250 were demultiplexed by sample and removed from their barcodes/adapters by the sequencing platform GenomeQc inc. Sequencing results have been made available on the NCBI website (NCBI—BioProject ID: PRJNA1099174, n.d.). On R studio version 4.2.2 (R Core Team 2022), the amplicon sequence variant (ASV) table was obtained using dada2 pipeline version 1.26 (Callahan et al. 2016) following tutorial version 1.16 (Callahan 2022) to trim, filter, merge, and remove chimeras from reads (Figures S2–S5). Thus, reads were filtered in pipeline as following: (1) as soon as the read had an occurrence of base quality lower than 2, it was truncated (truncQ argument); (2) For 16S, reads length exceeding 240 base pairs (bp) for the forward and 250 bp for the reverse were truncated, but since ITS reads tended to exceed the maximum read length of the MiSeq250 System, instead of being truncated like 16S, reads below 50 bp were discarded; (3) We set at 2 the maximum number of ‘expected errors’ allowed in a read; (4) Sequences with N were discarded (maxN = 0; Ambiguous base).

The taxonomy of ASV were assigned with Naive Bayesian Classifier algorithm (Wang et al. 2007) with a minimum bootstrap confidence set at 50, using the Silva database version 138.1 (McLaren and Callahan 2021) for 16S and the UNITE database version 8.3 (Abarenkov et al. 2021) for ITS, as training set. Finally, the functions of ASV (including trophic modes) were annotated using FunguildR for ITS and using MicFunPred for 16S (including KEGG ortholog groups), since these tools carry out a prediction with better or comparable accuracy and lower false‐positive predictions than other tools (e.g., PICRUST2, Piphilin or Tax4Fun2; Nguyen et al. 2016; Mongad et al. 2021).

Subsequently, the Phyloseq package version 1.42 was used to analyse communities and graphically display complex phylogenetic sequencing data (McMurdie and Holmes 2013). ASVs with unassigned high taxonomic rank are likely sequencing artefacts, as deep lineages (e.g., phylum, classes) are well represented in both taxonomic reference databases (Abarenkov et al. 2021; McLaren and Callahan 2021). Thus, 550 16S ASVs and 1126 ITS ASVs not assigned at the phylum level were removed. Then, 5289 bacterial ASVs and 867 fungal ASVs were also removed because they were rare (abundance threshold set at 15) or because they were weakly represented through the samples (prevalence threshold set at 5%; i.e., AVS must to be found in at least 3 samples) (Figure S6, Figure S7). We finally considered a total of 16,828 bacterial ASVs and 1328 fungal ASVs.

### 2.6 Statistical Analyses

To normalise uneven sequencing efforts, the depth of all samples has been reduced to the minimal depth of 95% and 97% of samples for 16S and ITS, respectively (i.e., 20,000 reads for 16S and 11,395 reads for ITS). Thus 5 samples with extremely low depth were removed (i.e., SA2 from initial sampling time and UC4 and JE1 from after salt sampling time). At this depth, samples passed the inflection point of the rarefaction curve (Figure S8) produced with ranacapa package on R (Kandlikar and Cowen 2018). To respect homoscedasticity, a variance‐stabilising transformation was performed using the DESeq2 package (Love et al. 2014). Analyses were stratified by sampling time to align with the study's objectives. Pre‐salt comparisons focused on differences between plant species in the absence of salt influence (Objectives 1 and 2), while post‐salt analyses assessed the effects of salt concentrations and their interaction with plant species on microbial communities (Objectives 3 and 4).

For alpha diversity analysis, ANOVA was performed to test whether the Shannon index (that consider richness and uniformity) differed significantly between sampling times, species, or NaCl concentrations. When significant, the post hoc test of Tukey HSD (Honest Significant Differences) comparisons was performed.

Considering the beta diversity, the differences between sampling times, species, or species * NaCl interaction were tested with a permutational multivariate analysis of variance (Adonis). When significant, post hoc tests were done using the PairwiseAdonis package (Martinez Arbizu 2020). Bray–Curtis dissimilarity was used as the distance metric to quantify compositional dissimilarities. Also, the multivariate homoscedasticity assumption for the Adonis test was systematically verified using a BetaDisper test. If significant, a permutation test was performed as a BetaDisper post hoc.

When the beta‐diversities were significatively different between plant species, the ASV differential abundance (ASV fold change between plant species) was analysed with the DESeq2 package (Love et al. 2014). Only highly significant *p* value adjusted with Benjamini & Hochberg procedure were considered (using 0.01 as False Discovery Rate cut‐off; Benjamini and Hochberg 1995). When the term ‘significant’ and related expressions were used, it refer to results of statistical tests with *p* values below 0.05. A summary of statistical test outcomes is provided in Table S4.

### 2.7 Linking Microbial Composition to System Performance

To explore potential relationships between microbial communities and BR performance, we performed redundancy analysis (RDA). RDA models were constructed separately for fungal genera and bacterial orders to explain variance in measured removal efficiencies of key contaminants that have been previously published in Beral et al. (2023b) and summarised in Table S8. Microbial taxa with high collinearity (Variance Inflation Factor > 10) were filtered or reduced through variable selection. Model significance was assessed using permutation tests (*n* = 999) for the global model, individual axes, and individual taxa. A table of top bacterial and fungal Spearman correlations with BR removal performance efficiency and RDA ordination is provided in Figure S11.

## 3 Results

We first characterised overall bacterial and fungal communities in BR, and then determined how planted species and salty runoff influenced their composition.

For the bacterial 16S, 6% of the reads were assigned at the species level, 38% at the genus level, 63% at the family level, 86% at the order level, and 96% at the class level (Figure S7). For the fungal ITS, the assignment is more efficient since 43% of reads were assigned at the species level and 89% at the genus level (Figure S7). For this reason, we will describe below the bacterial community at the taxonomic level of the order, while we will do it at the genus level for fungi.

Without considering the unassigned, the 16,828 bacterial ASV including 99 Archaea, represented 52 phyla, 137 classes, 274 orders, 346 families, 702 genera, and 418 species, when the 1328 fungal ASV covered 13 phyla, 45 classes, 96 orders, 212 families, and 365 genera (Table S5).

### 3.1 Dominant Bacteria and Fungi

**FIGURE 2:**
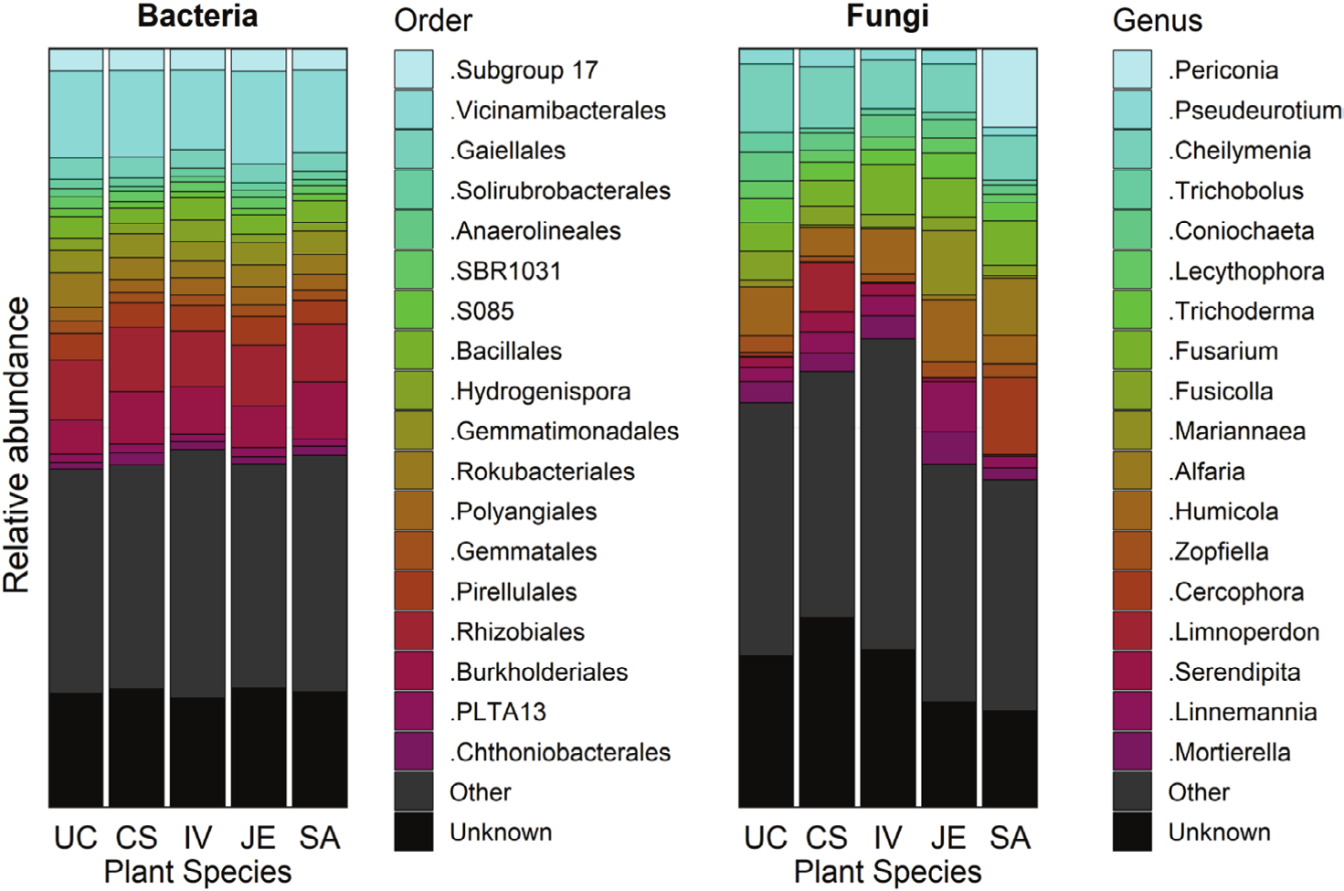
Top 20 of the most abundantly represented bacterial orders and fungal genera according to plant species used (CS: *Cornus sericea* , JE: *Juncus effusus* , IV: *Iris versicolor* , SA: *Sesleria autumnalis* ) or unplanted (UC).

**FIGURE 3:**
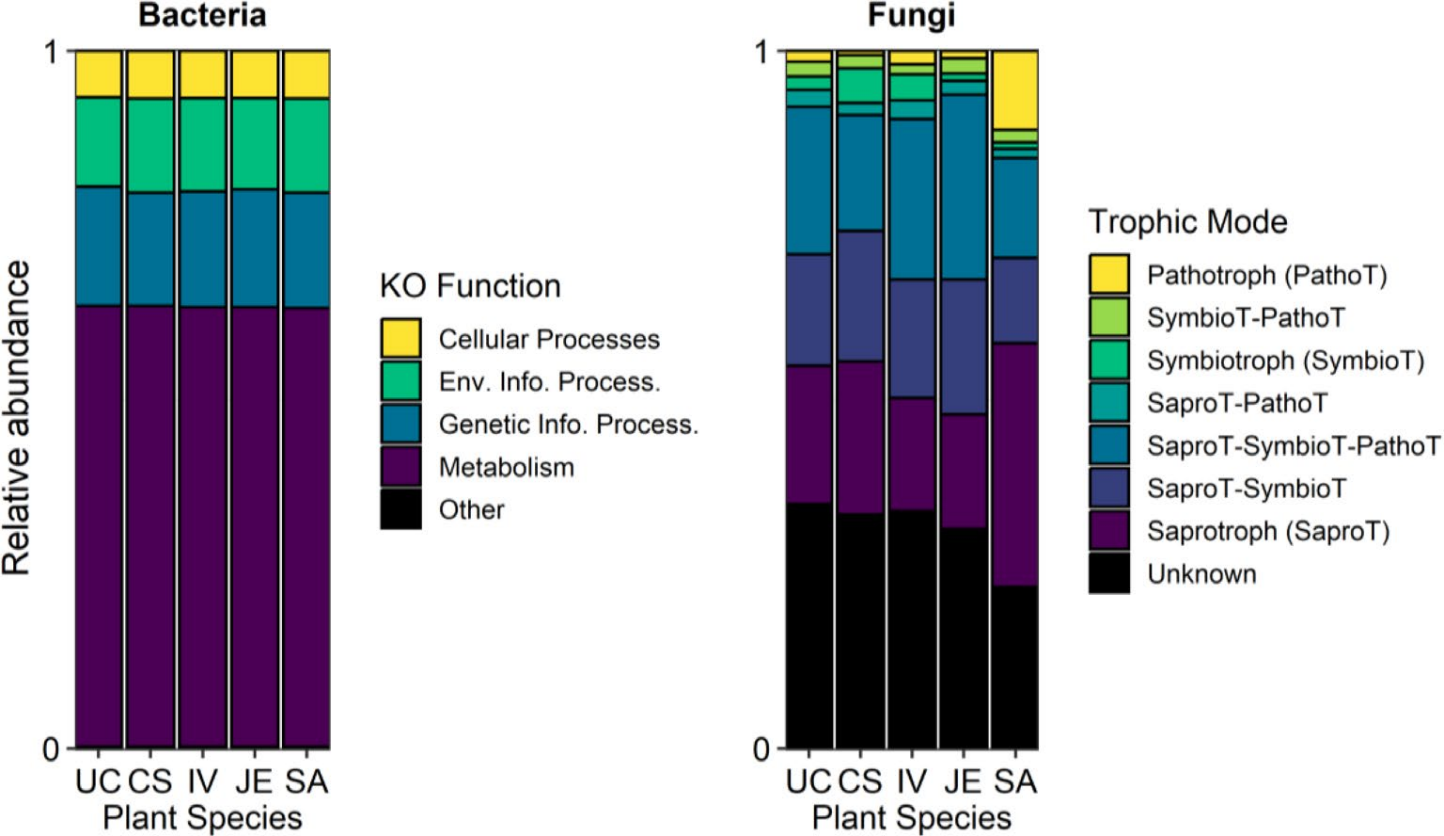
The proportion of each predicted bacterial function (Kegg orthologue) or fungal trophic modes according to the plant species used (CS: *Cornus sericea* , JE: *Juncus effusus* , IV: *Iris versicolor* , SA: *Sesleria autumnalis* ) or unplanted (UC).

BR mesocosms have been mostly colonised by bacteria and fungi that are ubiquitous in soil or thriving in moist environments such as aquifers, marine sediments, submerged wood, wetlands, sewage, and sludge, often contaminated with nitrogen and metals (Figures 2, S5, and S6). Some of the fungi known as psychrophiles dominated (present in the top 20) the mesocosm communities, probably due to their optimum growth temperatures below 20°C (Siddiqui et al. 2013). Psychrophilic taxa such as Vicinamibacterales, Humicola spp., and Mortierella spp. likely benefitted from the cold temperature conditions during sampling (approx. 10°C in March), suggesting environmental influence on community structure. We did attempt to detect the presence of taxa corresponding to the commercial inoculant in our sequencing data at the end of the experiment but found no clear signal. In our samples, metabolism involves more than half of the functions, and 2% of bacterial energy metabolism is devoted to the nitrogen cycle (Figure S9). More than half of the fungi in our BR systems exhibit a saprotrophic trophic mode, with some symbiotrophs also present but only a few pathotrophs (Figure 3).

Among the top 20 of the most represented bacterial orders (Figure 2), four can be involved in the nitrogen cycle (nitrogen‐fixing, nitrifying, denitrifying), the Rokubacteriales, the Pirellulales, but also the Burkholderiales well known to be able to nitrification, and heterotrophic denitrification (Prosser et al. 2014; Zielińska et al. 2016), and the Rhizobiales from which some of them are able to fix nitrogen in symbiosis with plant roots. Six orders (i.e., Solirubrobacterales, Bacillales, Rokubacteriales, Polyangiales, Burkholderiales, PLTA13) were found with probable or known metal tolerance or bioremediation functions (Mg, Mn, Pb, Zn), and one (i.e., Rhizobiales) using polycyclic aromatic compounds (PAHs) or toxic aromatic hydrocarbons as a carbon source (Table S6). Among the 20 most represented fungal genera (Figure 2), many were saprotrophs, denitrifiers or plant symbionts (Table S7). Symbionts ranged from avirulent endophytes promoting plant growth (i.e., *Periconia* spp., *Coniochaeta* spp., *Serendipita* spp.) to plant pathogens (i.e., *Coniochaeta* spp., *Fusarium* spp.). One fungal genus found can be virulent against other fungi (i.e., *Hormiactis* spp.). Also, some of the most represented fungal genera were known as psychrophiles (i.e., *Pseudallescheria* spp., *Humicola* spp., *Mortierella* spp.), or to tolerate high osmotic pressure (i.e., *Pseudallescheria* spp.; Table S7).

### 3.2 Plant Effect Before Application of Salty Runoff

In the first phase of the experiment, the effect of vegetation and differences between plant species on the diversity and composition of bacterial and fungal communities was studied before the application of salt runoffs.

**FIGURE 4:**
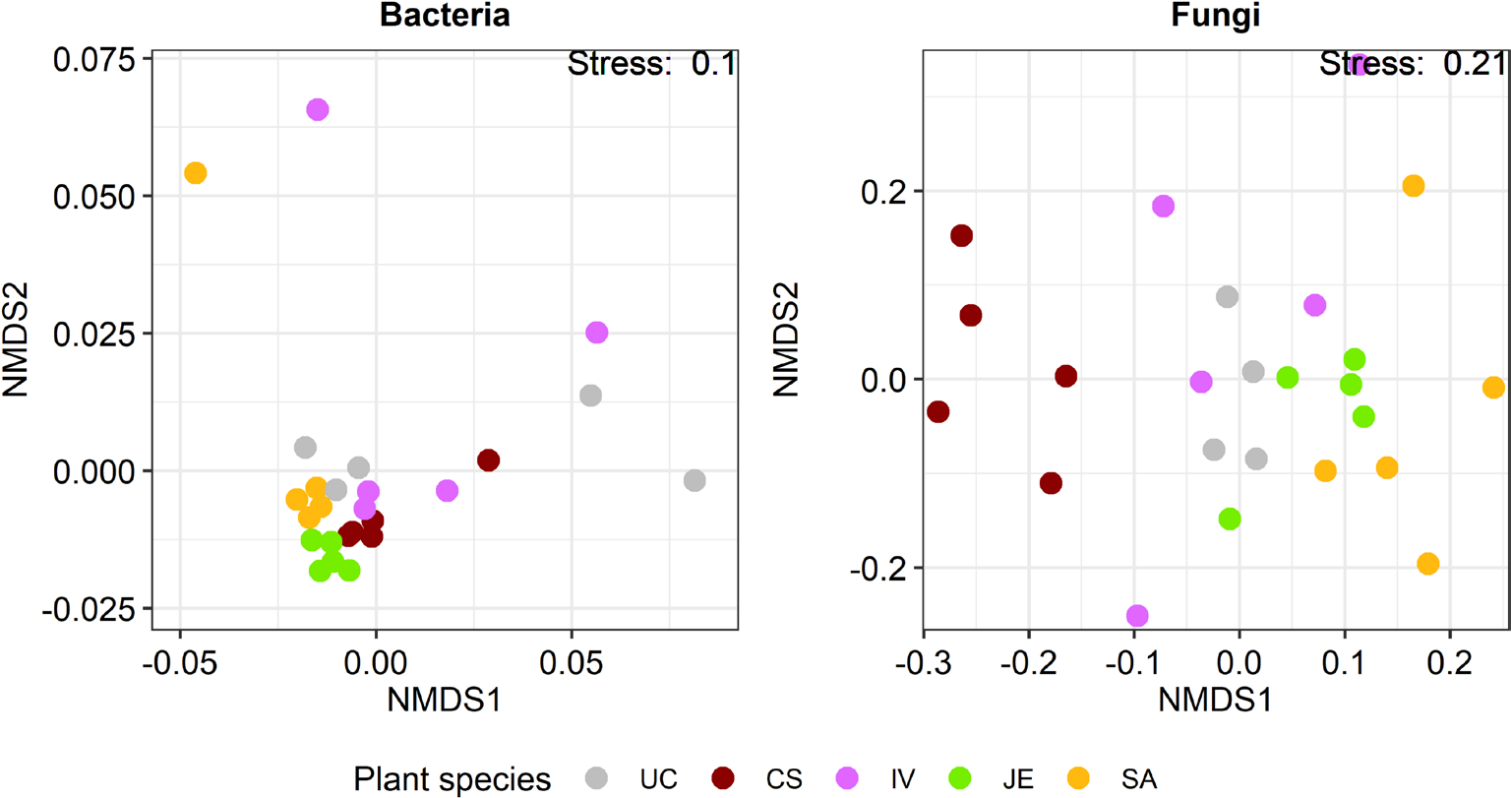
Non‐metric multidimensional scaling (nMDS) ordination of the bacterial (including archaea) and fungal beta diversity before spring salt runoffs using Bray and Curtis's method.

**FIGURE 5:**
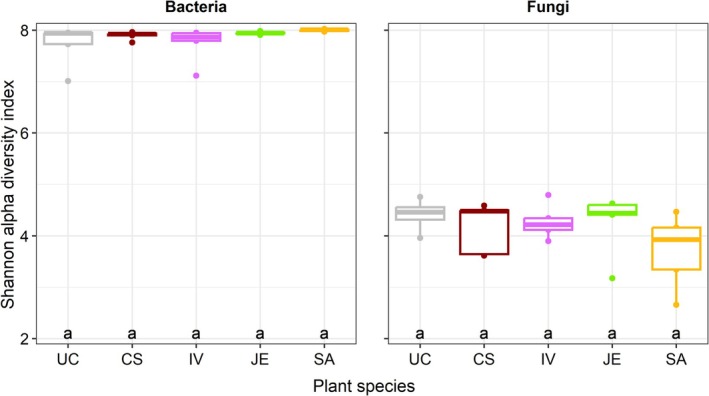
Bacterial (including archaea) and fungal Shannon alpha diversity index before spring salt runoff application (CS: *Cornus sericea* , JE: *Juncus effusus* , IV: *Iris versicolor* , SA: *Sesleria autumnalis* , UC: Unplanted).

Before spring salt runoffs, the bacterial (including archaea) communities (beta diversity) were significantly different between *Cornus* and *Juncus* (Figure 4). Concerning fungi, communities were significantly different between all plant species used. But this difference could have been partially explained by the dispersion within plant species groups (in other words, by the distance between individuals of the same species). This was the case for *Iris* with *Juncus*, and Unplanted with *Cornus*, *Iris*, and *Sesleria*. For both bacteria (including archaea) and fungi, the alpha diversity such as the Shannon index reflecting richness and evenness remained similar between the plant species used (Figure 5). As suspected, mesocosms planted with *Sesleria* harbour a greater abundance of pathotrophic fungi.

The microbial function prediction shows that the proportion of each bacterial function according to the Kegg orthologue classification appears to be quite similar for each plant species (Figure 3).

### 3.3 Differential Abundance Between Plant Species

The difference in bacterial and fungal communities observed according to plant species could be statistically explained by the differential abundance of certain ASV in these communities (Figure S10). These differences are summarised in Table 1. Compared to the other mesocosms, those planted with *Cornus* had significantly more bacteria of the orders Propionibacteriales and Chloroplast and more fungi of the genera *Limnoperdon* spp., *Serendipita* spp., *Pulvinula* spp., but less bacteria of the orders Nitrososphaerales and less fungi of the genera *Schizothecium* spp. Mesocosms planted with *Juncus* were characterised by significantly more bacteria of the orders Polyangiales and Micromonosporales, and less fungi of the genera *Aspergillus* spp. and *Mariannaea* spp. than other mesocosms. Mesocosms planted with *Juncus* have less abundant bacteria of the order Steroidobacterales than other mesocosms. However, this difference was not significant compared with *Cornus*. The mesocosms planted with *Iris* were found to have less bacteria of the orders Polyangiales, Vicinamibacterales, Sphingomonadales but much more bacteria of the orders Pseudomonadales, Rhizobiales, Micrococcales, Burkholderiales, Caulobacterales and Micromonosporales, and more fungi of the genera *Funneliformis* spp. than all other mesocosms. But this difference was not significant compared with *Cornus*. The mesocosms planted with *Sesleria* did not show any difference in differential abundance of bacteria when compared to those unplanted. Also, they presented more unknown bacteria. Compared to other mesocosms, they presented less bacteria of the orders Burkholderiales, Rhizobiales, Polyangiales and less or different ASV within Vicinamibacterales and Kineosporiales orders. Mesocosms planted with *Sesleria* presented significantly more fungi of the genera *Talaromyces* spp. and not assigned (NA), but a lower abundance of fungi of the genera *Serendipita* spp. and *Zopfiella* spp. than other mesocosms.

**TABLE 1:** Significantly more (+), less (−) or equal (=), abundant taxa for each species/unplanted compared to each other, as a result of the differential abundance test.

	Cornus	Juncus	Iris	Sesleria	Unplanted	
Bacteria						
Burkholderiales			+	− =Unplanted		
Caulobacterales			+			
Chloroplast	+					
Kineosporiales				−		
Micrococcales			+			
Micromonosporales		+				
Nitrososphaerales	−					
Polyangiales		+		− =Unplanted		
Propionibacteriales	+					
Pseudomonadales			+			
Rhizobiales			+	− =Unplanted		
Sphingomonadales			+			
Steroidobacterales		+ =Cornus				
Unknown				+ =Unplanted		
Vicinamibacterales			+ =Cornus	−		
Fungi						
Aspergillus		−				
Funneliformis			+ =Cornus			
Limnoperdon	+					
Mariannaea		−				
Pulvinula	+					
Schizothecium	−					
Serendipita	+			−		
Talaromyces				−		
Zopfiella				−		

### 3.4 NaCl Effect

In the second phase of the experiment, the salt experiment, the effect of plant species on the diversity and composition of bacterial and fungal communities was studied in interaction with 4 concentrations of de‐icing salt applied, approximately 5 months after the application of this salt‐laden runoff (Figure 1).

**FIGURE 6:**
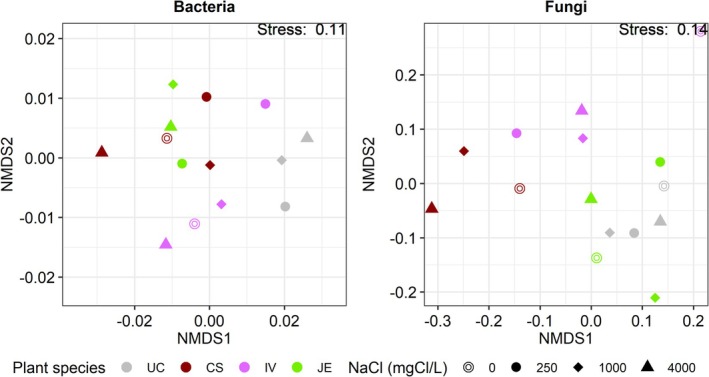
Beta diversity after spring salt runoffs application. Bacteria (including archaea) or Fungi non‐metric multidimensional scaling (nMDS) ordination using Bray and Curtis method; CS, *Cornus sericea* ; JE, *Juncus effusus* ; IV, *Iris versicolor* ; SA, *Sesleria autumnalis* ; UC, unplanted.

**FIGURE 7:**
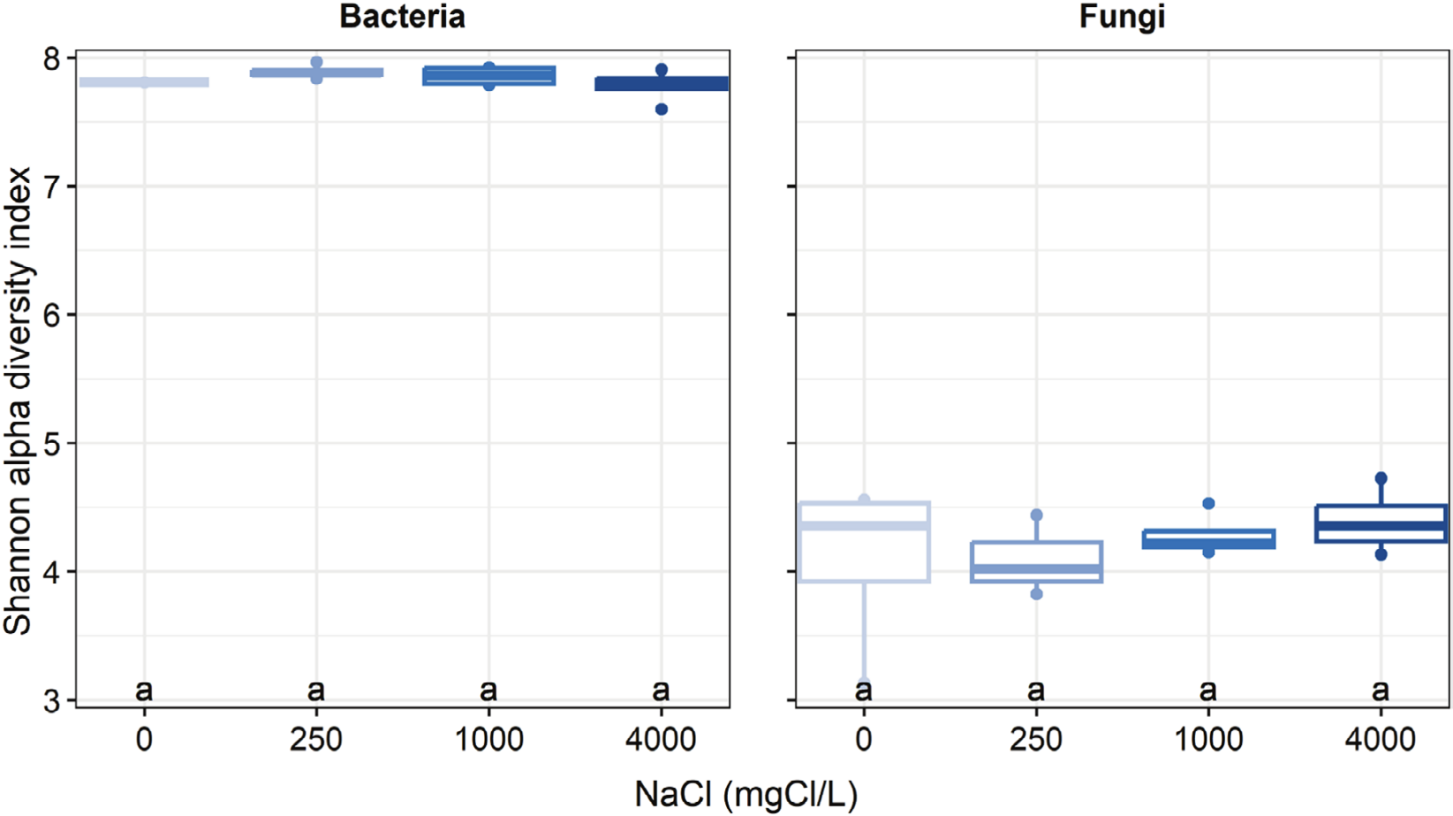
Bacterial (including archaea) and fungal Shannon alpha diversity index 5 months after spring salt runoffs at different NaCl concentrations.

For both Bacteria (including Archaea) and Fungi, no significant effect of spring salt runoffs was observed on communities (beta diversity), regardless of the concentration applied (Figure 6). It should be noted that for Bacterial communities, beta disperse test showed heterogeneity within NaCl groups that could have led to the non‐significance of the Adonis test. Without beta diversity difference found, it is thus without surprise that we did not find either any effect of spring salt runoffs on bacterial (including archaea) or fungal alpha diversity (Figure 7).

### 3.5 Relationships Between Microbial Taxa and Chemical Performance

RDA analysis revealed a statistically significant relationship between microbial community composition and BR performance (*p* = 0.015 for fungal; *p* = 0.016 for bacterial). Several bacterial orders were significantly associated with higher removal efficiency of nutrients or metals, notably Micrococcales, Frankiales, and Thermaerobacterales (*p* < 0.05). On the fungal side, Pseudeurotium, Pulvinulaceae, and Enterocarpus were among the top contributors (*p* < 0.05). These results suggest that specific microbial groups may be involved in processes enhancing BR contaminant removal. Visualisations of RDA results and a heatmap showing the top co‐variation between microbial taxa and removal metrics are provided in Figure S11.

## 4 Discussion

Although our experiment took place in a greenhouse, potentially limiting the entry of microorganisms, the experimental conditions (substrate, duration of the experiment, ventilation, use of semi‐synthetic water, supplemented rainwater from the roof of the greenhouse) allowed the microbial colonisation of mesocosms. In addition to the ubiquitous organisms found, BR mesocosms have been colonised by bacteria and fungi adapted to a moist and contaminated environment. This was expected since BR were watered to mimic an incoming volume of contaminated runoff from a comparatively large surface area. Although in this experiment no change in diversity level was caused by the presence of plants, the plant species significantly influenced the composition of the bacterial community observed, that is, the bacterial taxa and to a lesser extent the fungal taxa identified. These plants' recruitment could greatly influence the BR's performance. On the other hand, the application of de‐icing salt in a short period of time in spring was not enough to affect communities in any perceivable way toward the end of the growing season. Importantly, our RDA confirmed that these microbial communities are not only taxonomically distinct but they are also functionally relevant. Certain bacterial and fungal taxa were significantly associated with improved removal of nutrients and trace metals. This reinforces the importance of selecting plant species capable of shaping beneficial microbial communities and highlights the potential of vegetation as a biotic filter to optimise ecological processes within BR systems.

### 4.1 Dominant Bacterial and Fungal Taxa in BR Mesocosms

The microbial communities observed in our BR mesocosms closely resemble those found in a full‐scale BR located in Trois‐Rivières (Québec, Canada), which utilised the same substrate and plant species in polyculture (Dagenais et al. 2022). This similarity supports the transferability of our findings to operational BR functioning under real‐world conditions. More broadly, the microbial communities identified in our mesocosms reveal consistent patterns with those reported in other BR. Among the top 10 bacterial and archaeal phyla detected in our samples, 8 were also found to be dominant in BR studies by Zuo et al. (2019, 2020), Liu et al. (2020) and Fan et al. (2022), with additional overlap with Li et al. (2021), Chai et al. (2022) and Kong et al. (2022). This recurring presence of phyla suggests a broadly conserved microbial core across BR systems driven by characteristic BR conditions such as alternating flooding and drying, and exposure to runoff contaminants. Notably, many of these phyla have been implicated in key biogeochemical processes, including nitrogen cycling and contaminant degradation, reinforcing their potential functional significance in BR operation.

Functional inference based on the detected taxa indicates that the BR environment supports both aerobic and anaerobic microbial processes. The co‐occurrence of nitrifiers, denitrifiers, and anaerobic ammonium oxidizers suggests a microbial network capable of sustaining complete nitrogen transformation pathways. This aligns with previous studies that identified abundant nitrogen‐cycling genes in BRs and established correlations between key bacterial groups and nitrogen removal efficiency (Chen et al. 2013, 2019; Morse et al. 2018; Waller et al. 2018; Zuo et al. 2020). At the same time, the presence of nitrogen‐fixing bacteria highlights the potential for internally driven nitrogen inputs, which could either complement or compete with nitrogen removal depending on plant uptake and nitrogen availability of the substrate. The abundance of microbial taxa associated with metal tolerance and hydrocarbon degradation further points to the multifunctionality of BR systems. In line with findings from (Li et al. 2021; Goswami et al. 2022), the high relative abundance of these taxa suggests adaptation to contaminants commonly found in urban runoff and potential for in situ remediation. The detection of organohalide‐respiring bacteria implies that BR systems may also play a role in the attenuation of complex pollutants such as polychlorinated biphenyls (PCBs), as shown experimentally by Cao et al. (2021). In addition to this conserved microbial core, our mesocosms also supported site‐specific taxa likely shaped by local environmental conditions, such as psychrophilic or plant‐associated organisms.

Fungal communities similarly revealed both functional and ecological patterns. Many of the most represented orders and genera overlapped with those found in Canadian forest soils (Laperriere et al. 2019) and wetlands in the U.S. Midwest (Onufrak et al. 2020), suggesting environmental filtering or inoculation via mulch provided locally. Fungi likely contributed to substrate degradation, particularly saprotrophs capable of decomposing the fragmented ramial wood used in our BRs. This decomposition may influence nutrient availability and long‐term substrate performance. The presence of potential denitrifying fungi supports the idea that fungi can complement bacterial nitrogen removal, although their specific activity was not directly tested.

While several fungal genera found can form mutualistic associations with plants, others are known plant pathogens. The balance between beneficial and detrimental fungal interactions may influence plant survival and thus the overall performance of BRs. Interestingly, mycorrhizal inoculants added at planting were no longer detected 18 months later, suggesting that the persistence of introduced fungi may be limited compared to recruitment from native sources.

Altogether, our results emphasise that BR microbiomes are shaped by a combination of environmental conditions, substrate inputs, and ecological selection pressures. The recurring detection of key bacterial and fungal groups across studies and climates highlights the potential for designing BRs that leverage naturally assembling microbial communities for enhanced contaminant removal and system resilience.

### 4.2 Plant Effect on Bacterial and Fungal Communities

The constituent elements of the BR, such as the substrate or the type of runoff (frequency, volume, and contamination level) seem to explain only partially the bacterial and fungal communities present. Our results contrast with the findings of Brodsky et al. (2019). These authors reported lower alpha diversity in unplanted bioswale systems. Our unplanted mesocosms exhibited alpha diversity levels comparable to those of the planted systems, irrespective of the plant species used. However, the differences in beta diversity observed in our study reveal the importance of plant species choice in the composition and abundance of certain taxa within these communities. Thus, although the unplanted mesocosm communities share most of the dominant taxa with the vegetated ones, the plant species *Cornus*, *Juncus*, and *Iris* had certain specific orders/genera (Rhizobiales, Burkholderiales, *Serendipita* spp., Zopfiella spp.) in the soil. Moreover, these plant‐recruited taxa have functions that are very useful for BR performance, such as involvement in the nitrogen cycle or promoting plant growth. *Cornus* recruited numerous *Serendipita* spp. fungi, known to establish ectomycorrhizal symbioses with many plant families, and may also have been beneficial for *Cornus* (Table S7). The Rhizobiales were significantly more present in mesocosms planted with *Iris*. Although the literature does not mention Rhizobiales symbiosis with *Iris versicolor* , few nodules have been observed on the roots when dismantling the mesocosms. Shurigin et al. (2022) observed bacterial endophytes from *Bacillus* spp. in *Iris pseudacorus*, a species closely related to *Iris versicolor* . Species of the genus Bacillus are also known to form nodules. *Bacillus megaterium* strain NMp082 was isolated from *Medicago polymorpha* (Chinnaswamy et al. 2018). Rhizobiales are described as nitrogen‐fixing bacteria that could have potentially decreased the nitrogen removal efficiency of BRs planted with *Irises*. *Iris* communities had also more Burkholderiales (including nitrifiers and denitrifiers) than other species. It is therefore difficult to know which way the balance will tip between nitrogen fixation or removal in that case. It is also important to note that these conclusions are based on functional inference from taxonomic presence alone, and without transcriptomic validation, these conclusions remain speculative. In general, it is not recommended to choose plant species that can establish nitrogen‐fixing symbiosis, at the risk of threatening the BR performance. The mesocosms planted with *Sesleria* presented, on the contrary, significantly less Burkholderiales in their communities and also had lower nitrogen removal efficiency (Beral et al. 2023b).

Brodsky et al. (2019) noticed that plant species with higher transpiration rates were associated with higher alpha and beta diversity. Even though the plants tested in our experiment covered different functional traits (such as transpiration rate, roots morphology, physiology; see Beral et al. (2023b)), we did not observe a consistent relationship between these traits and microbial alpha diversity when comparing average diversity values per species. However, due to the limited number of plant species included, the variation in traits was not sufficient to allow a statistical analysis of potential trait–microbiome relationships.

### 4.3 Salt Effect on Bacterial and Fungal Communities

Knowing all the consequences that de‐icing salt can have on micro‐organisms, it was quite surprising not to observe any lasting effects of these salt treatments on the bacterial (including archaea) and fungal communities toward the end of the growing season, whatever the concentration applied with salt runoff in spring.

The absence of detectable long‐term effects of salt application on microbial community structure is most plausibly attributed to community recovery during the five‐month interval between salt exposure and sampling. Although the applied concentration (up to 4000 mg Cl^−^/L) may cause acute toxicity, the high solubility and mobility of chloride ions, combined with the open‐drainage design of the BR mesocosms, likely facilitated rapid leaching of salts from the substrate. Effluent monitoring confirmed the absence of detectable Cl^−^ concentrations 1 month after application (Beral et al. 2023a), suggesting limited duration of microbial exposure. Furthermore, the microbial taxa present prior to salt addition included few halotolerant species (Table S7), and electrical conductivity remained consistently low in all inflows before treatment (20 ± 3 μS·cm^−1^), indicating no prior acclimation to salinity. While transient impacts on microbial structure cannot be ruled out, the delayed sampling may have masked short‐term effects. Future studies employing higher temporal resolution and repeated pulse exposures would be better suited to distinguish immediate microbial responses from subsequent recovery dynamics.

## 5 Conclusion

Our findings reveal that in our experiment, BR mesocosms were naturally colonised by microbial communities well‐adapted to cold, humid, and contaminated conditions, characteristic of urban runoff in temperate climates. These communities include taxa involved in essential ecological functions that could be useful in improving BR cells performance, such as nitrogen cycling, metal tolerance, hydrocarbon degradation, and symbiosis with plants.

Beyond this broadly shared microbial core, our findings revealed that plant species exert a strong selective influence on microbial community composition. While richness and evenness (alpha diversity) remained relatively stable across both vegetation and salt exposure treatments, beta diversity analyses showed that microbial community structure varied significantly with plant identity. In particular, plant species such as *Cornus sericea* , *Iris versicolor* and *Juncus effusus* were associated with distinct bacterial and fungal taxa, some of which are known to perform key ecological functions such as nitrogen fixation (*Rhizobiales*), denitrification (*Burkholderiales*), or plant symbiosis (*Serendipita* spp.). These results highlight the important role of vegetation as a biotic filter in BR cells design, shaping the microbial communities that underpin many essential biogeochemical processes. While the commercial inoculant that may have been beneficial during the initial stages of plant establishment, its lack of long‐term persistence raises questions about its relevance in BR systems. This reinforces the importance of plant species selection to more sustainably shape microbial communities over time.

Short‐term exposure to de‐icing salt at realistic runoff concentration had no detectable long‐term effect on either bacterial or fungal community structure in BR. Transient effects may have occurred, and the delayed sampling likely masked them. However, this suggests a degree of resilience among BR microbial communities to episodic salinity disturbances. Future studies with higher temporal resolution are needed to capture short‐term impacts.

Taken together, our results highlight three key insights. First, plant species selection plays a central role in shaping microbial communities, beyond the establishment of a shared microbial core. Second, because commercial inoculants did not persist long term, plant selection appears to be a more sustainable lever for optimising microbial functions in BR systems. Third, microbial communities showed resilience to short‐term salinity exposure, supporting the robustness of BR systems in climates with seasonal de‐icing salt inputs. To build on these findings, future studies should combine microbial community profiling with direct measures of functional activity and explore the potential use of substrate from mature BR systems as inoculum for new installations.

In conclusion, this study contributes to the growing recognition that microbial ecology plays a fundamental role in green infrastructure design. By explicitly linking plant identity to microbial community composition and testing resilience to realistic urban stressors, we provide new evidence to guide the development of more robust, ecologically informed BR systems. Future studies should also explore the potential of using substrate from older BR systems as inoculants for new installations, as these could offer organisms well‐suited to specific system conditions, potentially accelerating their ecological function and stability.

## Author Contributions

**Henry Beral:** conceptualization, writing – original draft, writing – review and editing, formal analysis, investigation, data curation. **Jacques Brisson:** conceptualization, writing – review and editing, supervision, funding acquisition. **Margit Kõiv‐Vainik:** conceptualization, writing – review and editing, supervision, funding acquisition. **Joan Laur:** writing – review and editing. **Danielle Dagenais:** conceptualization, writing – review and editing, supervision, funding acquisition.

## Conflicts of Interest

The authors declare no conflicts of interest.

## Supporting information


**Data S1:** Supporting information.

## Data Availability

The data that supports the findings of this study are available in the Supporting Information of this article.
