# Supplementary material for: Influence of Plant Species and De‐Icing Salt on Microbial Communities in Bioretention
Source: Environ Microbiol Rep. 2025 Sep 7;17(5):e70193. doi: 10.1111/1758-2229.70193 (PMC12414797; doi:10.1111/1758-2229.70193)
Supplement: Supplementary file 1 — Data S1: Supporting information. [file EMI4-17-e70193-s001.docx]

Supplementary material

Influence of plant species and de-icing salt on microbial communities in bioretention

Henry Beral^1,^ *, Jacques Brisson^1^, Margit Kõiv-Vainik^1,2^, Joan Laur^1^, Danielle Dagenais^3^

1. Institut de recherche en biologie végétale, Département de sciences biologiques, Université de Montréal, 4101 East Sherbrooke St, Montréal, Québec, Canada, H1X2B2 ; henry.beral@umontreal.ca,
2. Institute of Ecology and Earth Sciences, University of Tartu, Vanemuise 46, 51003, Tartu, Estonia; margit.koiv.vainik@ut.ee
3. École d’urbanisme et d'architecture de paysage, Faculté de l’aménagement, Université de Montréal, 2940, chemin de la Côte-Sainte-Catherine, P.O. Box 6128, Downtown Station, Montréal, Québec, Canada, H3C3J7 ; danielle.dagenais@umontreal.ca

* Correspondence: [henry.beral@umontreal.ca](mailto:henry.beral@umontreal.ca)

## Tables

[Table S1. Soil characteristics before runoffs (average of soil cores at -10 and -30cm depth, analyzed in composite per species) and after runoffs (from a soil core at -10 depth), in mg/kg except when other units are displayed. 3](#_Toc204386215)

[Table S2. Semi-synthetic runoff composition without salt treatment: “Targeted” values correspond to the average quality of the runoff water according to Duncan, (1999). The values marked with “*” are non-targeted pollutants, present along with chemical sources added to. “Measured” values correspond to averages from analyses conducted monthly or at each watering. 4](#_Toc204386216)

[Table S3. PCR primer from “Earth Microbiome Project” (2022). 5](#_Toc204386217)

[Table S4. Summary of statistical test outcomes. 5](#_Toc204386218)

[Table S5. Bacterial and fungal proportion of read and ASV assigned at each taxonomic level. 5](#_Toc204386219)

[Table S6. Known functions and ecology of the 20 most represented Bacteria Order, sorted by general relative abundance. 6](#_Toc204386220)

[Table S7. Known functions and ecology of the 20 most represented fungi genus, sorted by general relative abundance. 7](#_Toc204386221)

[Table S8. Summary of bioretention mesocosms removal efficiency (Average removal pourcentage compare to the influent) across plant species and experimental phases. Data correspond to the same experimental phases as microbial community sampling, and were obtained from Beral et al. (2023a; 2023b). 8](#_Toc204386222)

Table S1. Soil characteristics before runoffs (average of soil cores at -10 and -30cm depth, analyzed in composite per species) and after runoffs (from a soil core at -10 depth), in mg/kg except when other units are displayed.

| Sampling time | **Before salt runoffs** | | | | **After salt runoffs** | | | | | | | | | | | | | | | |
| --- | --- | --- | --- | --- | --- | --- | --- | --- | --- | --- | --- | --- | --- | --- | --- | --- | --- | --- | --- | --- |
| Species | **Unplanted** | **C. sericea** | **I. versicolor** | **J. effusus** | **Unplanted** | | | | **C. sericea** | | | | **I. versicolor** | | | | **J. effusus** | | | |
| NaCl concentration | **0** | | | | **0** | **250** | **1000** | **4000** | **0** | **250** | **1000** | **4000** | **0** | **250** | **1000** | **4000** | **0** | **250** | **1000** | **4000** |
| pH | 7.3 | 7 | 7.2 | 7.2 | 7.2 | 6.9 | 7.2 | 7.2 | 7 | 6.8 | 6.8 | 6.6 | 7 | 7 | 7.1 | 6.9 | 6.9 | 6.9 | 7.1 | 6.8 |
| Organic matter (%) | 6.8 | 6.9 | 7.2 | 7.1 | 6.2 | 6.7 | 6.7 | 6.6 | 8.4 | 6.6 | 7.8 | 8 | 5.9 | 6.3 | 7.1 | 6.5 | 6.6 | 6.9 | 7.2 | 6.8 |
| Conductivity (mS/cm) | 0.1 | 0.2 | 0.2 | 0.2 | 0.1 | 0.4 | 0.3 | 0.1 | 0.1 | 0.2 | 0.2 | 0.2 | 0.1 | 0.1 | 0.1 | 0.1 | 0.1 | 0.3 | 0.5 | 0.2 |
| CEC (meq/100g) | 15.3 | 15.8 | 14.8 | 15.3 | 17.1 | 18.1 | 17.4 | 14.8 | 17.9 | 18.5 | 18.4 | 21.5 | 17.2 | 17.1 | 15.9 | 17.3 | 17.6 | 17.6 | 18.4 | 18.6 |
| SAR | 1.2 | 1.1 | 1.4 | 1.4 | 1.1 | 0.9 | 0.7 | 1.2 | 1.2 | 1.9 | 1.1 | 1.3 | 1.4 | 1.6 | 1.8 | 2.2 | 1.1 | 1.5 | 3.8 | 1.6 |
| Na | 66 | 63 | 76 | 77 | 67 | 52 | 45 | 66 | 70 | 111 | 61 | 79 | 83 | 95 | 103 | 127 | 67 | 84 | 233 | 93 |
| Cl | 18 | 17 | 34 | 73 | 18 | 11 | BDL | 17 | 48 | 73 | 20 | 35 | 16 | 17 | 16 | 19 | 23 | 73 | 214 | 81 |
| Ca | 6157 | 5807 | 5915 | 6110 | 6973 | 6271 | 6937 | 6039 | 6648 | 6333 | 6013 | 6578 | 6719 | 6429 | 6489 | 6120 | 6454 | 6283 | 7218 | 6527 |
| Mg | 380 | 379 | 378 | 383 | 383 | 451 | 466 | 331 | 464 | 480 | 410 | 509 | 292 | 381 | 363 | 380 | 337 | 294 | 350 | 356 |
| Mn | 112 | 184 | 133 | 122 | 137 | 140 | 139 | 143 | 144 | 124 | 143 | 136 | 123 | 142 | 187 | 158 | 141 | 123 | 156 | 241 |
| TN | 1800 | 2300 | 2200 | 2100 | 2100 | 2000 | 2200 | 1900 | 2600 | 2300 | 1900 | 2200 | 1900 | 2500 | 2300 | 2100 | 2000 | 2300 | 2300 | 2300 |
| NO2-NO3 | 7 | BDL | BDL | 3 | 3 | 59 | 21 | 3 | BDL | BDL | BDL | BDL | BDL | BDL | BDL | BDL | BDL | BDL | BDL | BDL |
| P | 273 | 213 | 234 | 250 | 262 | 288 | 289 | 260 | 212 | 230 | 226 | 239 | 210 | 220 | 202 | 197 | 248 | 226 | 249 | 228 |
| K | 145 | 231 | 204 | 237 | 121 | 136 | 153 | 83 | 268 | 246 | 292 | 407 | 91 | 120 | 68 | 107 | 86 | 204 | 446 | 281 |
| Al | 5720 | 5850 | 5030 | 5490 | 6940 | 5440 | 5950 | 6310 | 6480 | - | - | 5060 | 6000 | 6370 | 5800 | 5820 | 5960 | 4290 | 5930 | 5720 |
| Cu | 12 | 19 | 14 | 17 | 16 | 18 | 33 | 16 | 26 | 19 | 27 | 16 | 17 | 20 | 17 | 26 | 21 | 25 | 20 | 26 |
| Fe | 8720 | 9570 | 7410 | 8410 | 11100 | 9050 | 9920 | 10500 | 9680 | - | - | 8920 | 9210 | 8970 | 9120 | 9050 | 8880 | 6430 | 9810 | 8790 |
| Zn | 30 | 37 | 35 | 30 | 46 | 37 | 39 | 42 | 44 | 33 | 39 | 36 | 41 | 38 | 47 | 49 | 36 | 33 | 38 | 44 |
| **As, B, Cd, Cr, Co, Ni, Pb were below detection limit (BDL)** | | | | | | | | | | | | | | | | | | | | |

Table S2. Semi-synthetic runoff composition without salt treatment: “Targeted” values correspond to the average quality of the runoff water according to Duncan, (1999). The values marked with “*” are non-targeted pollutants, present along with chemical sources added to. “Measured” values correspond to averages from analyses conducted monthly or at each watering.

| **Parameter** | **Source** | **Targeted** | **Measured** |
| --- | --- | --- | --- |
| B (ppm) | Calcimax | 40.9* | - |
| C (ppm) | C_6_H_12_O_6_ | 20.0 | 43.0 |
| Ca (ppm) | CaCl_2_ + Calcimax | 32.4 | 31.6 |
| Cl (ppm) | CaCl_2_ | 0.677* | - |
| Cr (ppm) | KCr(SO_4_)2·12(H_2_O) | 0.060 | 0.060 |
| Cu (ppm) | CuSO_4_ | 0.095 | 0.109 |
| Fe (ppm) | EDDHA-FeNa | 5.0 | 5.3 |
| K (ppm) | K_2_SO_4_ | 8.6 | 10.3 |
| Mg (ppm) | MgSO_4_ | 0.450 | 1.58 |
| Mn (ppm) | EDTA-MnNa_2_ | 0.020 | 0.130 |
| Mo (ppm) | Na_2_MoO_4_ | 0.010 | - |
| Ni (ppm) | NiSO_4_·6 (H_2_O) | 0.050 | 0.052 |
| NOx (ppm) | NH_4_NO_3_ | 5.5 | 10.3 |
| P (ppm) | KH_2_PO_4_ | 0.4 | 0.4 |
| Pb (ppm) | Pb (NO_3_)_2_ | 0.350 | 0.341 |
| S (ppm) | K_2_SO_4_ | 4.15* | - |
| Zn (ppm) | ZnSO_4_ | 0.450 | 0.563 |
| pH |  |  | 6.9 |
| TDS (ppm) |  |  | 447 |
| DO (%) |  |  | 57.9 |
| DO (ppm) |  |  | 5.6 |

Table S3. PCR primer from “Earth Microbiome Project” (2022).

| Organisms | Amplicon | Primer | Reference | Sequence |
| --- | --- | --- | --- | --- |
| Bacteria | 16S | 515F | Parada et al., 2016 | GTGYCAGCMGCCGCGGTAA |
|  |  | 806R | Apprill et al., 2015 | GGACTACNVGGGTWTCTAAT |
| Fungi | ITS | ITS1f | Gardes and Bruns, 1993 | CTTGGTCATTTAGAGGAAGTAA |
|  |  | ITS2 | White et al., 1990 | GCTGCGTTCTTCATCGATGC |

Table S4. Summary of statistical test outcomes.

| **Organisms** | **Diversity** | **Period** | **Betadisper** | **P-value** |
| --- | --- | --- | --- | --- |
| 16S | Alpha | Before salt - plant effect |  | 0.298 |
| 16S | Beta | Before salt - plant effect | 0.170 | 0.001 |
| ITS | Alpha | Before salt - plant effect |  | 0.333 |
| ITS | Beta | Before salt - plant effect | 0.006 | 0.001 |
| 16S | Alpha | After salt - salt effect |  | 0.263 |
| 16S | Beta | After salt - salt effect | 0.002 | 0.077 |
| ITS | Alpha | After salt - salt effect |  | 0.702 |
| ITS | Beta | After salt - salt effect | 0.767 | 0.887 |

| **Beta diversity: Before salt - plant effect** | | | | | | |
| --- | --- | --- | --- | --- | --- | --- |
|  | | **ITS** | | | | |
|  |  | **CS** | **IV** | **JE** | **SA** | **UC** |
| **16S** | **CS** | - | 0.007 | 0.010 | 0.008 | 0.006 |
|  | **IV** | 0.007 | - | 0.011 | 0.025 | 0.022 |
|  | **JE** | 0.010 | 0.014 | - | 0.006 | 0.007 |
|  | **SA** | 0.007 | 0.346 | 0.008 | - | 0.014 |
|  | **UC** | 0.014 | 0.095 | 0.006 | 0.107 | - |

Table S5. Bacterial and fungal proportion of read and ASV assigned at each taxonomic level.

| Taxonomic level | ASV assigned | ASV unassigned | read assigned (%) |
| --- | --- | --- | --- |
| 16S | | | |
| Class | 137 | 798 | 96% |
| Order | 274 | 2453 | 86% |
| Family | 346 | 5594 | 63% |
| Genus | 702 | 10426 | 38% |
| Species | 418 | 16196 | 6% |
| ITS | | | |
| Class | 45 | 35 | 99% |
| Order | 96 | 94 | 95% |
| Family | 212 | 148 | 91% |
| Genus | 365 | 200 | 89% |
| Species | 349 | 850 | 43% |

Table S6. Known functions and ecology of the 20 most represented Bacteria Order, sorted by general relative abundance.

| **Top 20** | **Function and ecology** |
| --- | --- |
| **Bacteria Order** |  |
| **Subgroup 17** |  |
| **Vicinamibacterales** | aerobic, neutrophilic, psychrophilic to mesophilic chemoheterotrophs (Huber and Overmann, 2018) |
| **Gaiellales** | Especially the aerobic *Gaiella* Genus that has been also found in Portugal aquifers (Albuquerque *et al.*, 2011). |
| **Solirubrobacterales** | Especially the families 67-14 and Solirubrobacteraceae that are often associated to bioremediation of Pb and Zn (Goswami *et al.*, 2022). |
| **Anaerolineales** | Anaerolineae are usually found in marine sediment (Breuker *et al.*, 2011). |
| **SBR1031** |  |
| **S085** |  |
| **Bacillales** | Especially A. pallidus species with a potential application in bioremediation (Harirchi *et al.*, 2020). |
| **Hydrogenispora** | Previously isolated from an anaerobic sludge treating herbicide wastewater (Liu *et al.*, 2014). |
| **Gemmatimonadales** | Especially the *Gemmatimonas* Genus. The first member of this genus was discovered in 2003 in sewage treatment sludge (Zhang *et al.*, 2003). |
| **Rokubacteriales** | Neutrophilic non-methanotrophic bacteria that can colonize nitrogen-rich wetlands (Ivanova *et al.*, 2021). We identified some Rokubacteriales from the WX65 Familly that have been previously found to be positively correlated with both Mn and Zn presence (Goswami *et al.*, 2022). |
| **Polyangiales** | Negatively correlated with Zn presence (Goswami *et al.*, 2022). |
| **Gemmatales** | Largely represented with the Fimbriiglobus, Gemmata and *Zavarzinella* Genus. Part of the Planctomycetes Phyllum, which are often able of anaerobic ammonium oxidation. |
| **Pirellulales** | Part of the Planctomycetes Phyllum, which are often able of anaerobic ammonium oxidation (anammox). Represented with numerus Genus from the Pirellulaceae familly such as the *Pirellula*, or the *Pir4 lineage*. Pirellula spp. are cosmopolite and play a role in global carbon and nitrogen cycles (ScienceDirect Topics). |
| **Rhizobiales** | Represented among others by the Xanthobacteraceae, Hyphomicrobiaceae families. The Rhizobiales thrive in freshwater and wet soil. Some of them are able to fix nitrogen in symbiosis with plant roots (especially under reduced O2 pressure) (Delgado *et al.*, 2007; Stacey, 2007) or are chemolithoautotrophic and can use polycyclic aromatic compounds (PAHs) or toxic aromatic hydrocarbon such as naphthalene as the sole carbon source (An *et al.*, 2013; Herbst *et al.*, 2013). |
| **Burkholderiales** | Represented among others with Nitrosomonadaceae and Comamonadaceae Families. Burkholderiales are known to be often able of nitrification, and heterotrophic denitrification (Prosser *et al.*, 2014; Zielińska *et al.*, 2016). They have often been isolated from soil, sludge, water and industrial environments or mining dumps. They are one of the main genus of bacteria associated with fungal hyphae, even in "pristine" environments. |
| **PLTA13** | Previously reported in many caves (Bastian *et al.*, 2009; Bogdan *et al.*, 2023) and in the biofilm microbial community of a Mn-rich mine from Sweden in which they have a potential implication in Mg removal (Sjöberg *et al.*, 2020). |
| **Chthoniobacterales** | Represented among others with the Chthoniobacteraceae and the Xiphinematobacteraceae families. |

Table S7. Known functions and ecology of the 20 most represented fungi genus, sorted by general relative abundance.

| Top 20  Fungi Genus | Function and ecology |
| --- | --- |
| Periconia | Primarily know as root colonizing endophytes (Jumpponen et al., 2017) and plant pathogen (Gunasekaran *et al.*, 2021). |
| Pseudeurotium | Only represented with the species P. hygrophilum that can thrive in a diverse environments (Russian montane fen soil; Northern Finland peatlands rich in arsenic sulphate, and nitrate; Antarctica substrates; associated with sponges) (Ogaki *et al.*, 2020). |
| Cheilymenia | Cosmopolitan species |
| Coniochaeta | Often tree pathogens (Damm *et al.*, 2010) and some species can be endophytic mycorrhizal (Harrington *et al.*, 2019). |
| Lecythophora | Only represented by L. canina species. First discovered as an opportunistic dogs infectious agent, causing canine osteomyelitis (Troy *et al.*, 2013). |
| Trichoderma | Soil ubiquists. Many species are opportunistic avirulent plant symbionts, protecting plants from parasites and antagonists of many phytopathogenic fungi (Harman and Kubicek, 2002a, 2002b; Harman, 2006; Vinale *et al.*, 2008; Brotman *et al.*, 2010; Hermosa *et al.*, 2012). Some are able of denitrification (Mothapo *et al.*, 2015). |
| Fusarium | Phytopathogenic and sometimes saprophytic (Wollenweber, 1931; Ma *et al.*, 2013). Some are able of denitrification (Mothapo *et al.*, 2015). |
| Fusicolla | Only represented by F. aquaeductuum species. Previously found in sewage (Steensland, 1973). |
| Enterocarpus | Only represented by E. grenotii species. |
| Pseudallescheria | Only represented by P. boydii species. that cause many human diseases in temperate climates (Cortez *et al.*, 2008). This species is thermotolerant and also tolerate minimal aeration and high osmotic pressure, thus observed in nitrogen rich environments, generally related to human activity (Cooke and Kabler, 1955; Guarro *et al.*, 2006) |
| Humicola | Psychrophiles and saprotroph of coniferous and deciduous forest soils (Kubicek, 2007; Zhang *et al.*, 2021). |
| Zopfiella | Only represented by Z. lundqvistiis species know to growth on submerged wood (Shearer and Crane, 1978). |
| Cercophora | Only represented by C. fici species. |
| Hormiactis | Uncommon fungi, known as a soil saprophyte that could also infect fungi, causing them a cap spotting disease (Agriculture Victoria, 2021). |
| Auricularia | Saprotrophic wood-rotters that generate white rot (Worrall *et al.*, 1997). |
| Serendipita | Sebacinales Order is well know for their symbioses with >65 plant family (Oberwinkler *et al.*, 2013; Lee and Hawkes, 2021). |
| Linnemannia | Only represented with L. amoeboidea and L. hyaline species. |
| Mortierella | Typically saprobes in soil, dung, and reproductive bodies of higher fungi and facultative parasites (Zhang *et al.*, 2021). Many are psychrophiles and able to promote plant growth in agricultural soils (Li *et al.*, 2018; Ozimek and Hanaka, 2021). |

Table S8. Summary of bioretention mesocosms removal efficiency (Average removal pourcentage compare to the influent) across plant species and experimental phases. Data correspond to the same experimental phases as microbial community sampling, and were obtained from Beral et al. (2023a; 2023b).

| **Removal (%)** | **TOC** | **TP** | **TN** | **NH4** | **NO3** | **Ca** | **Cd** | **Cr** | **Cu** | **Fe** | **K** | **Mg** | **Mn** | **Ni** | **Pb** | **Zn** |
| --- | --- | --- | --- | --- | --- | --- | --- | --- | --- | --- | --- | --- | --- | --- | --- | --- |
| **Before salt** | | | | | | | | | | | | | | | | |
| **C. sericea** | -3 | -15 | 24 | -77 | -85 | -2 | -6 | -318 | -52 | -13 | -90 | -31 | -116 | -49 | -76 | -46 |
| **I. versicolor** | -9 | -85 | -10 | -85 | -250 | 3 | -8 | -457 | -89 | -22 | -52 | -30 | -103 | -82 | -117 | -68 |
| **J. effusus** | 5 | -20 | 23 | -113 | -62 | 17 | 45 | -389 | -51 | -7 | 34 | -4 | -34 | -64 | -178 | -55 |
| **S. autumnalis** | -20 | -138 | -65 | -253 | -1320 | 10 | 17 | -528 | -99 | -23 | -229 | -17 | -56 | -135 | -322 | -129 |
| **Unplanted** | -15 | -174 | -148 | -106 | -2522 | 0 | -34 | -403 | -104 | -31 | -272 | -28 | -2 | -93 | -232 | -102 |
| **After salt** | | | | | | | | | | | | | | | | |
| **C. sericea** | -4 | 44 | 44 | -2 | 71 | -104 | -62 | -117 | -33 | -3 | -41 | -117 | -114 | -82 | -104 | -64 |
| **I. versicolor** | -27 | -15 | 7 | -83 | -33 | -131 | -90 | -191 | -79 | -29 | -45 | -153 | -441 | -110 | -124 | -89 |
| **J. effusus** | -4 | 52 | 45 | 4 | 80 | -85 | 11 | -100 | -27 | -8 | 55 | -104 | 42 | -47 | -71 | -14 |
| **S. autumnalis** | -33 | -35 | 9 | -143 | -30 | -144 | -16 | -196 | -47 | -29 | -122 | -167 | -375 | -98 | -200 | -57 |
| **Unplanted** | -54 | -135 | -105 | -170 | -356 | -153 | -157 | -312 | -93 | -53 | -234 | -190 | -335 | -169 | -397 | -215 |

## Figures

[Figure S1 : A. Schematic plan of the experimental layout of the 16 mesocosms in the greenhouse as "Latin square" configuration. The color and acronyms indicate plant species, and the blue border intensity indicates the level of salinity applied during the runoffs; B. Schematic of the mesocosm design; C. Picture of the 16 mesocosms in the IRBV greenhouse, 28 July 2020, Beral H.. 11](#_Toc204376590)

[Figure S2. 16S and ITS forward and reverse reads quality profile of the 67 aggregated samples, before or after filtering. 12](#_Toc204376591)

[Figure S3. 16S and ITS forward and reverse reads error rates, for each possible transition. Points are the observed error rates for each consensus quality score. The black line shows the estimated error rates after convergence of the machine-learning algorithm. The red line shows the error rates expected under the nominal definition of the Q-score. 13](#_Toc204376592)

[Figure S4. Number of 16S and ITS reads passed through each stage of the pipeline for each sample. 14](#_Toc204376593)

[Figure S5. 16S and ITS ASVs length frequency. 15](#_Toc204376594)

[Figure S6. A. 16S and B. ITS ASVs prevalence by abundance in each phylum (colors). The dotted lines represent the threshold of the applied filters. 17](#_Toc204376595)

[Figure S7. A. 16S and B. ITS ASVs number according to their abundance or prevalence, as well as their proportion of reads assigned by taxonomic level before or after filtering. 19](#_Toc204376596)

[Figure S8. 16S and ITS rarefaction curve per sampling period. The red dashed line represents the maximum depth applied to filtered samples. Colors indicate plant species (CS: *Cornus sericea*, JE: *Juncus effusus*, IV: *Iris versicolor*, SA: *Sesleria autumnalis*, UC: unplanted), and labels indicate replicates. 20](#_Toc204376597)

[Figure S9. Bacterial energy metabolism. 21](#_Toc204376598)

[Figure S10. 2 to 2 comparisons between planted species, of significantly different bacterial Order or fungal Genus’s abundances, before saline runoff; CS: *Cornus sericea*, JE: *Juncus effusus*, IV: *Iris versicolor*, SA: *Sesleria autumnalis*, UC: unplanted. 22](#_Toc204376599)

[Figure S11. Redundancy Analysis (RDA) and table of top bacterial and fungal Spearman correlations with BR removal performance efficiency. 25](#_Toc204376600)

**A
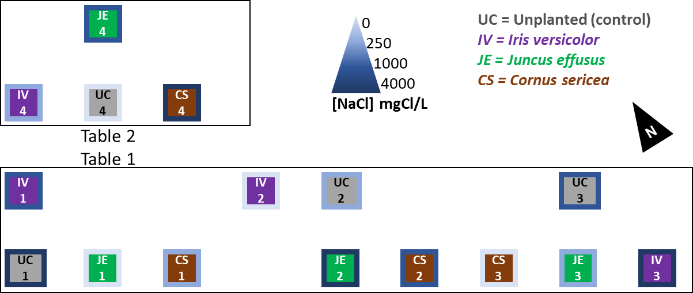
**

**B
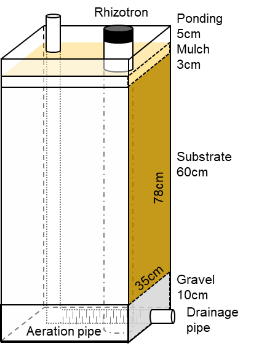
 C**
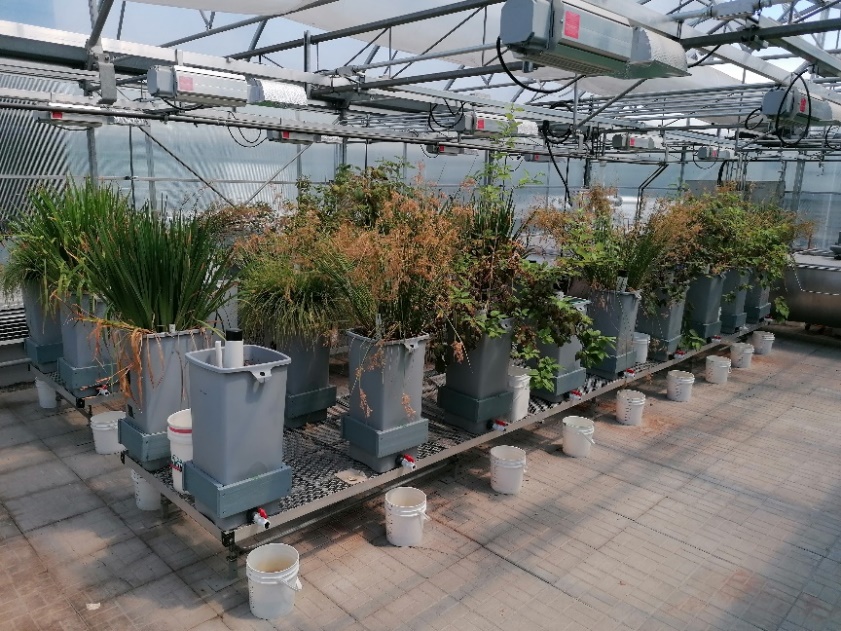


Figure S1 : A. Schematic plan of the experimental layout of the 16 mesocosms in the greenhouse as "Latin square" configuration. The color and acronyms indicate plant species, and the blue border intensity indicates the level of salinity applied during the runoffs;
B. Schematic of the mesocosm design;
C. Picture of the 16 mesocosms in the IRBV greenhouse, 28 July 2020, Beral H..


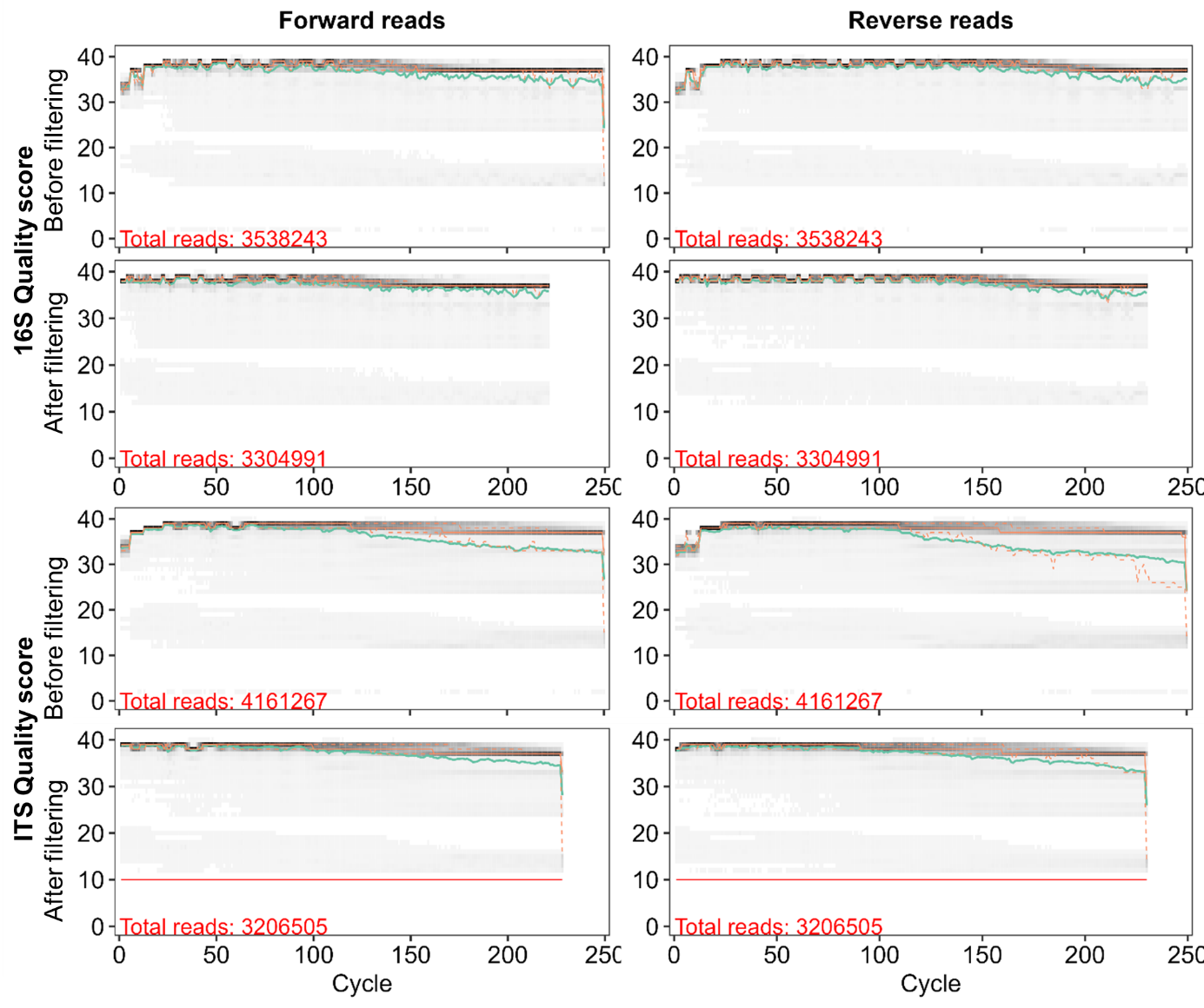


Figure S2. 16S and ITS forward and reverse reads quality profile of the 67 aggregated samples, before or after filtering.


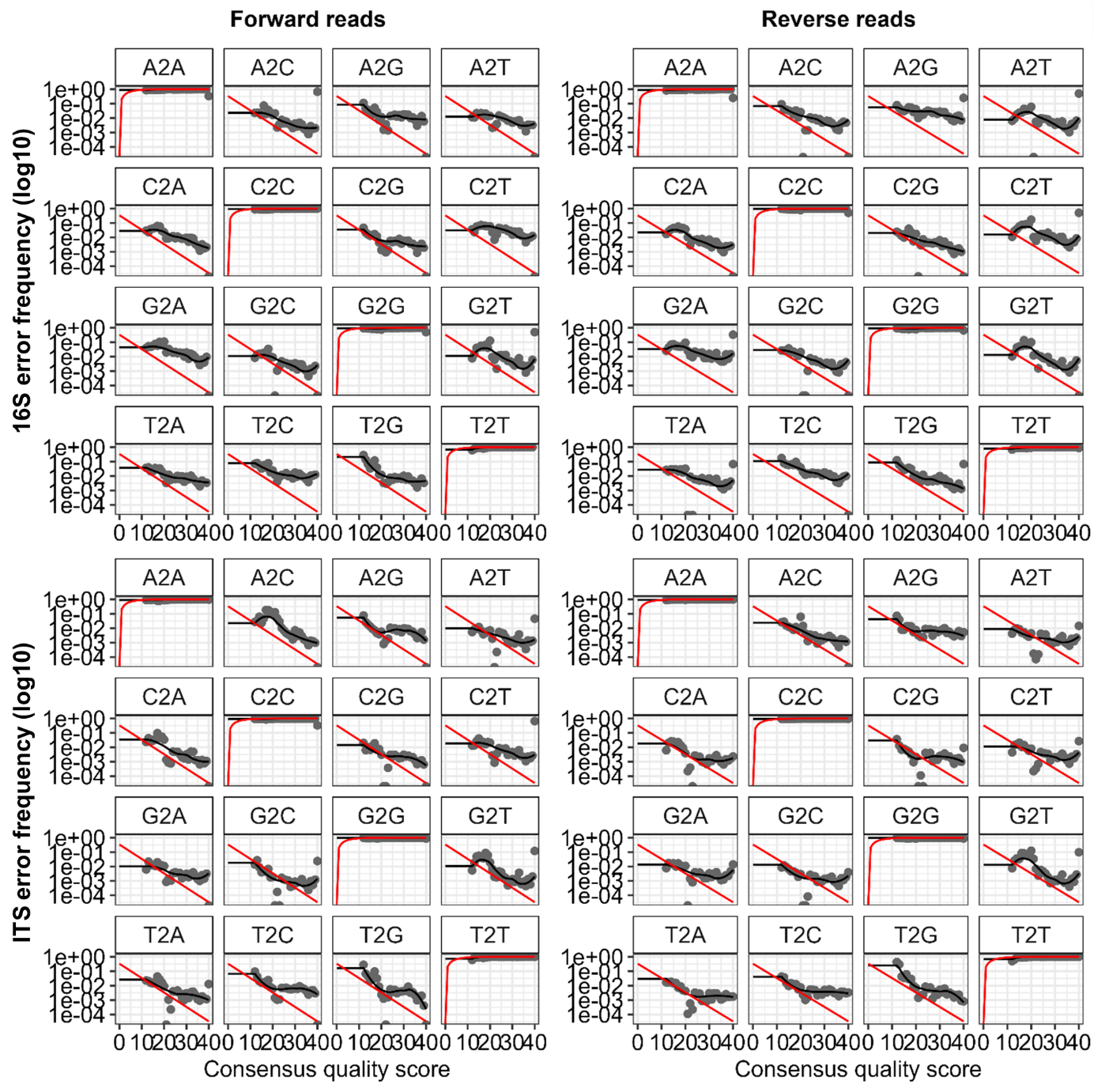


Figure S3. 16S and ITS forward and reverse reads error rates, for each possible transition.
Points are the observed error rates for each consensus quality score. The black line shows the estimated error rates after convergence of the machine-learning algorithm. The red line shows the error rates expected under the nominal definition of the Q-score.


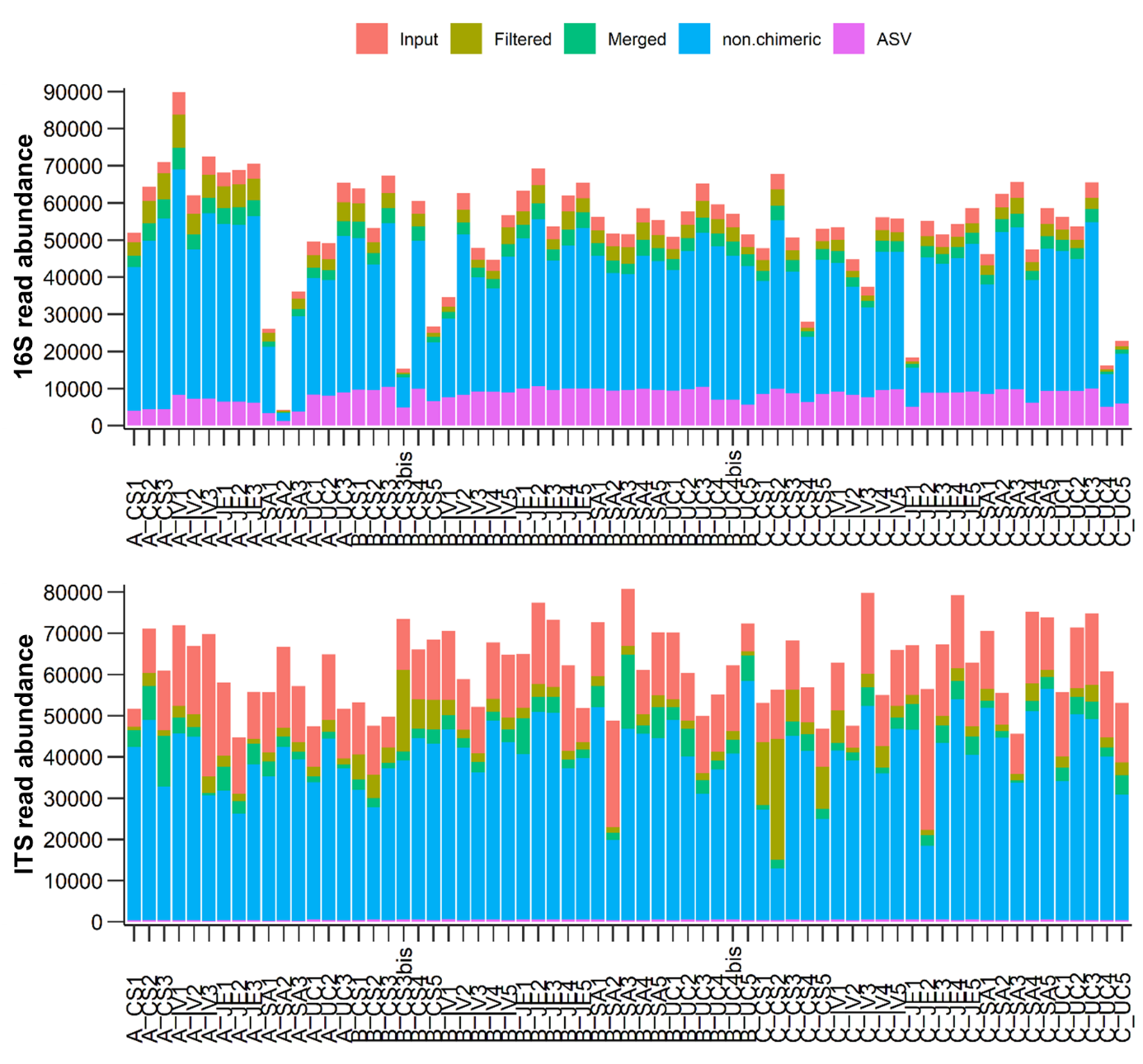


Figure S4. Number of 16S and ITS reads passed through each stage of the pipeline for each sample.


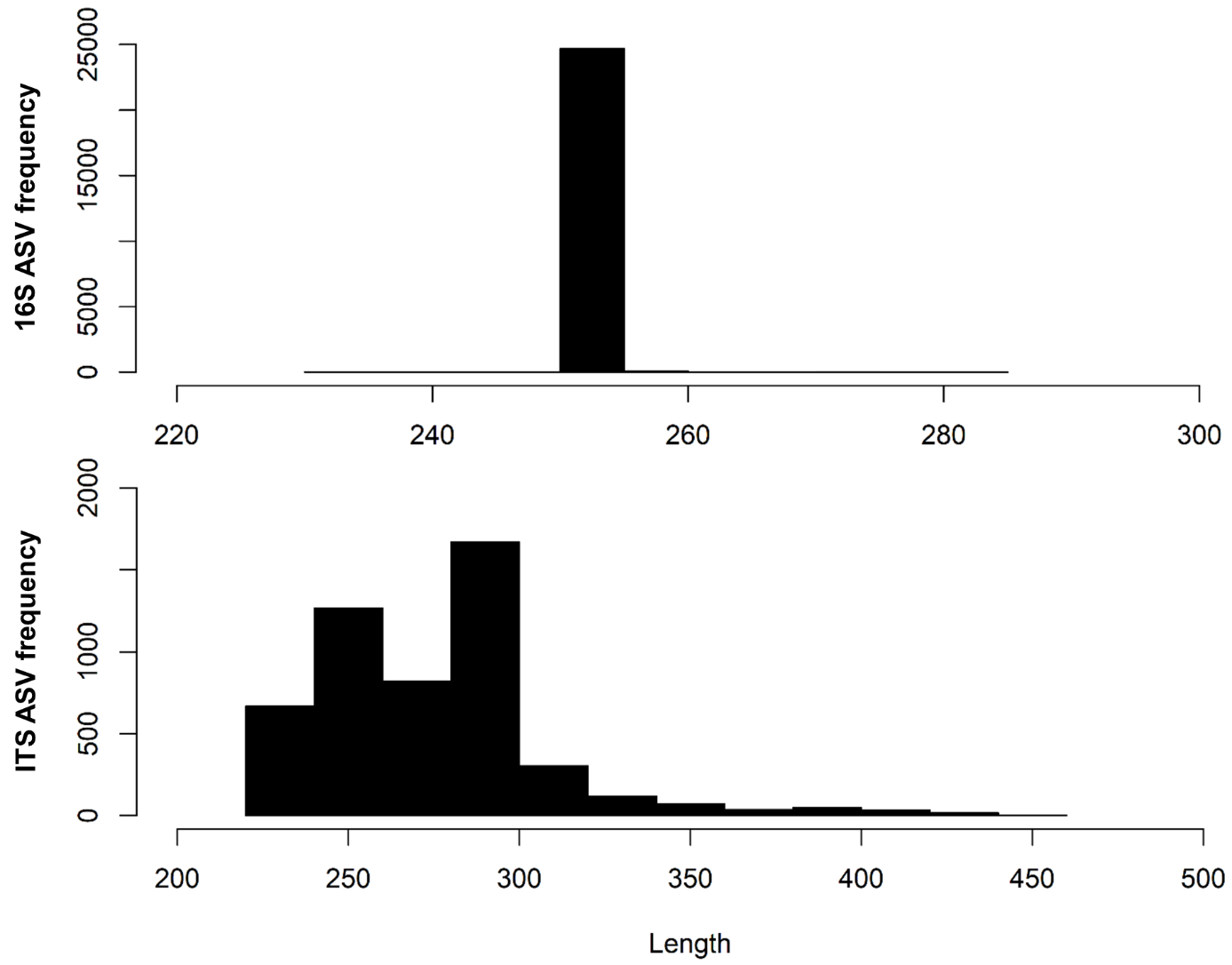


Figure S5. 16S and ITS ASVs length frequency.

**A**
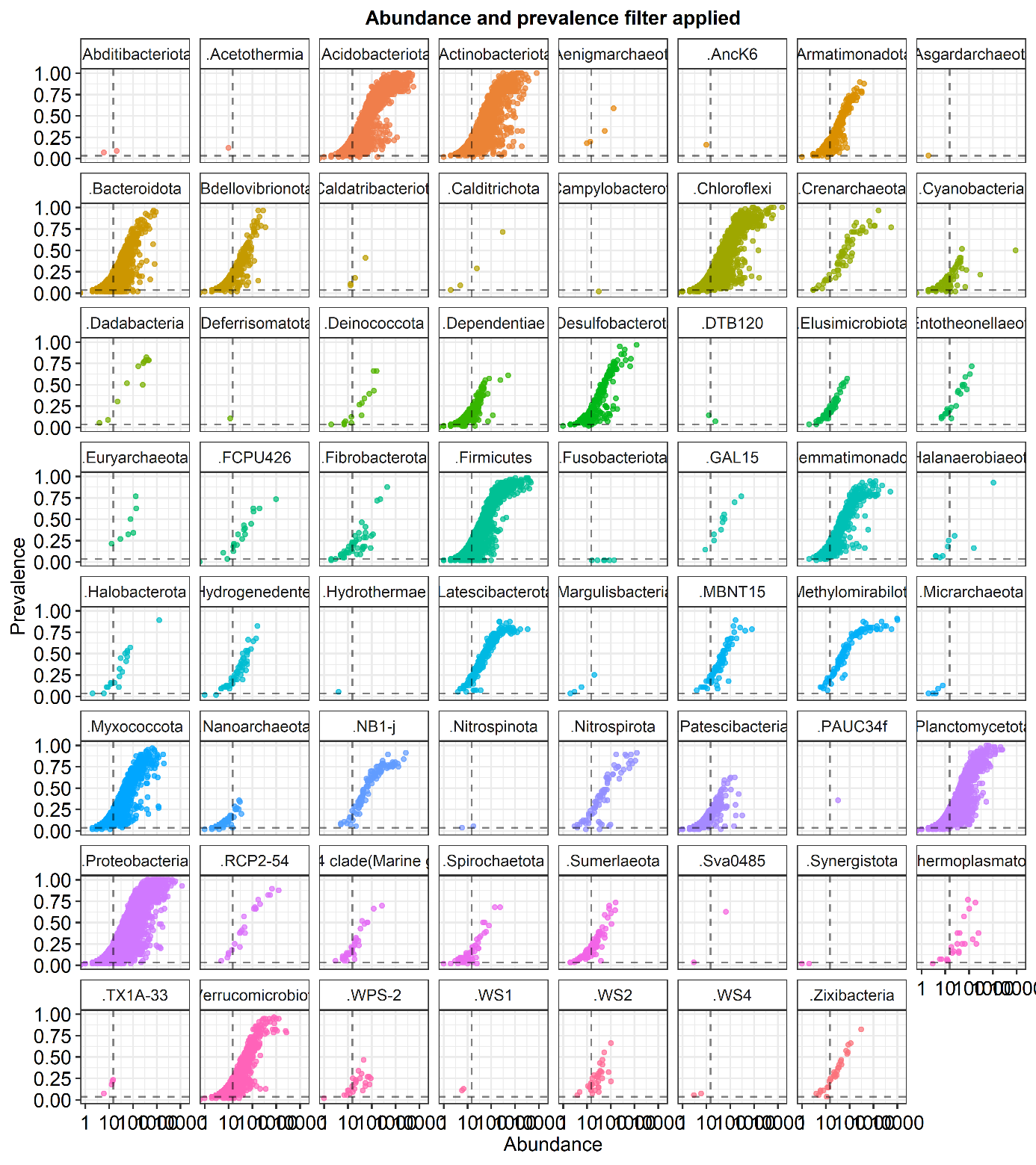


**B**
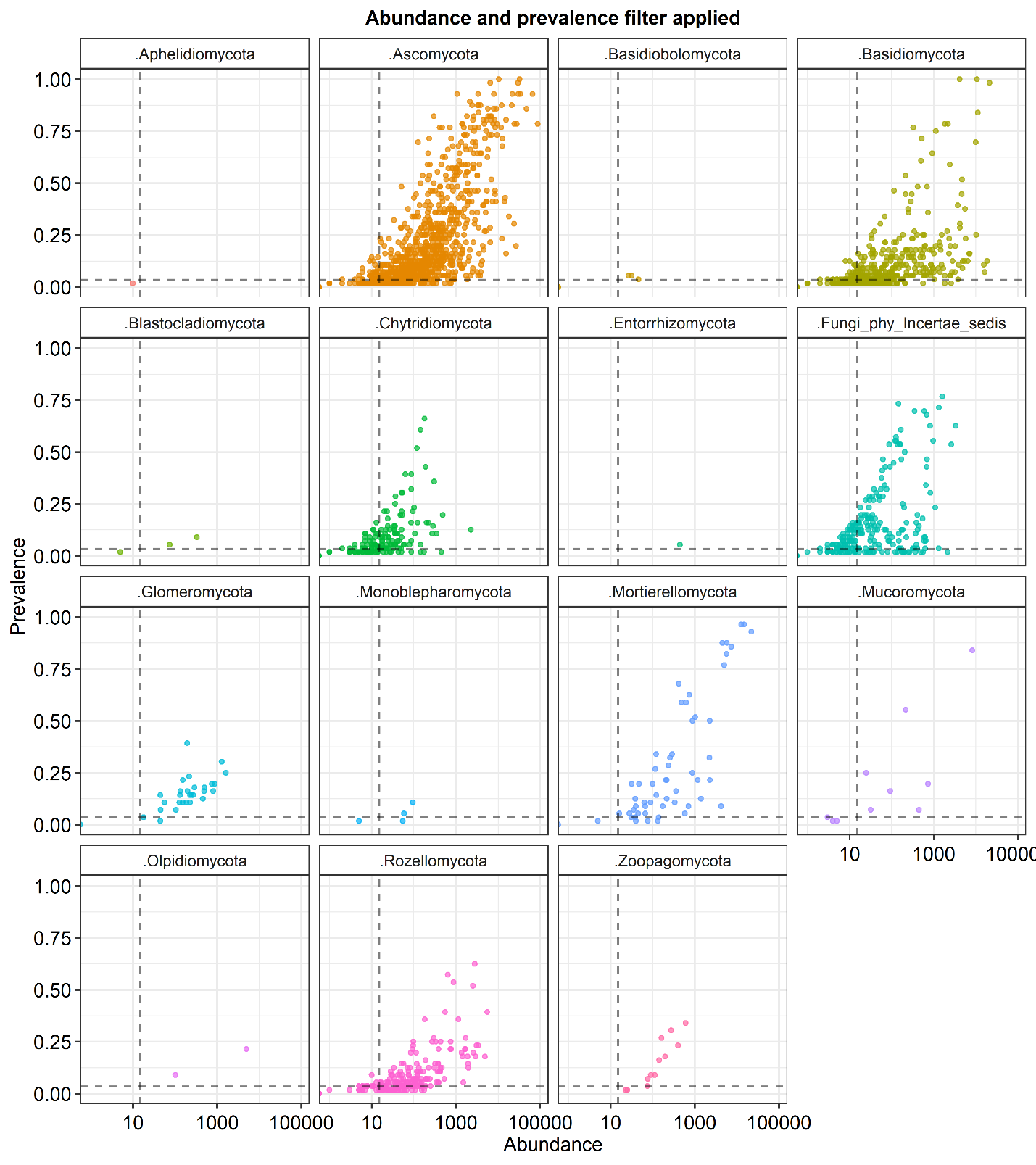


Figure S6. A. 16S and B. ITS ASVs prevalence by abundance in each phylum (colors).
The dotted lines represent the threshold of the applied filters.

**A**
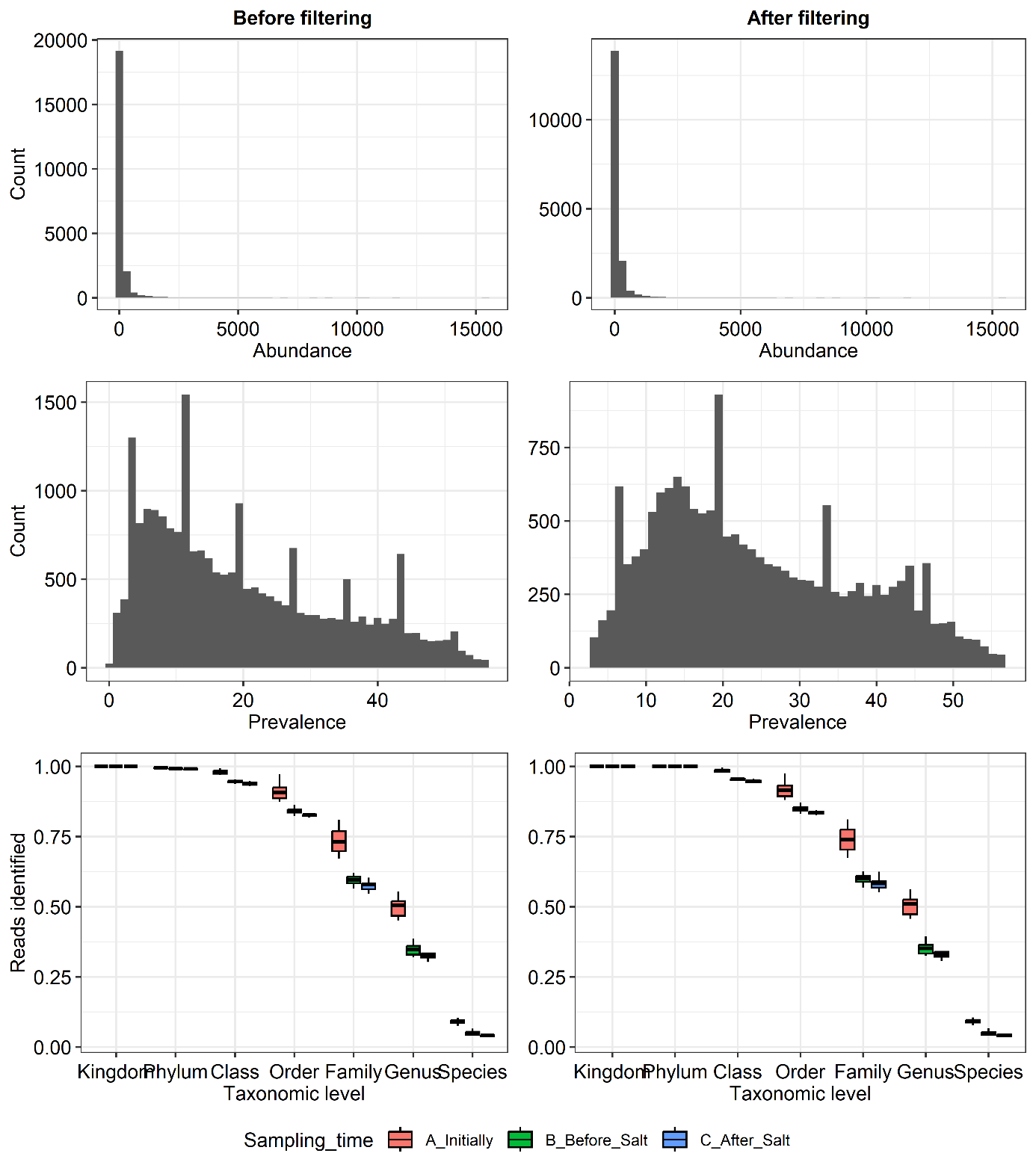


**B**
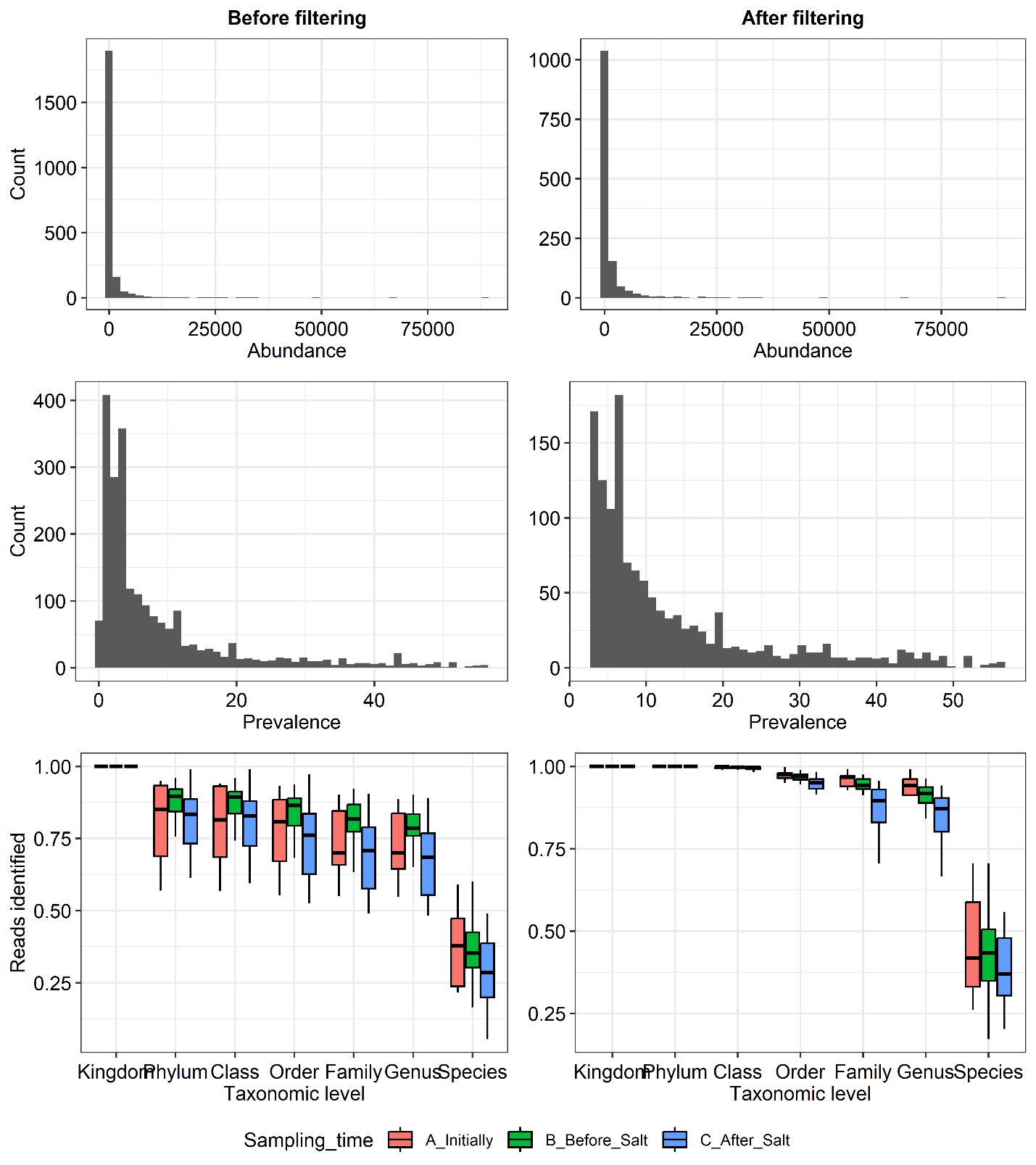


Figure S7. A. 16S and B. ITS ASVs number according to their abundance or prevalence, as well as their proportion of reads assigned by taxonomic level before or after filtering.


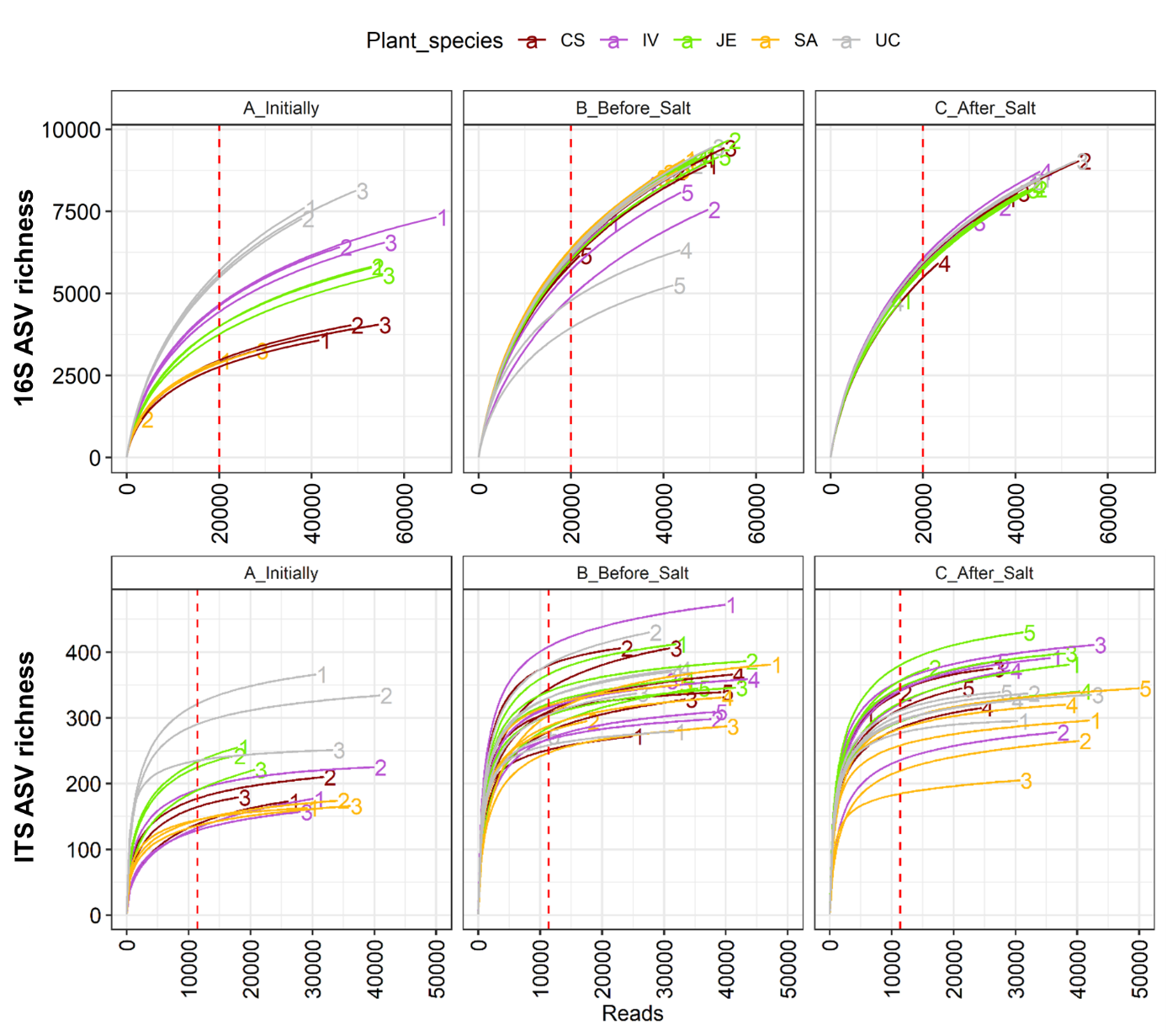


Figure S8. 16S and ITS rarefaction curve per sampling period.
The red dashed line represents the maximum depth applied to filtered samples. Colors indicate plant species (CS: *Cornus sericea*, JE: *Juncus effusus*, IV: *Iris versicolor*, SA: *Sesleria autumnalis*, UC: unplanted), and labels indicate replicates.


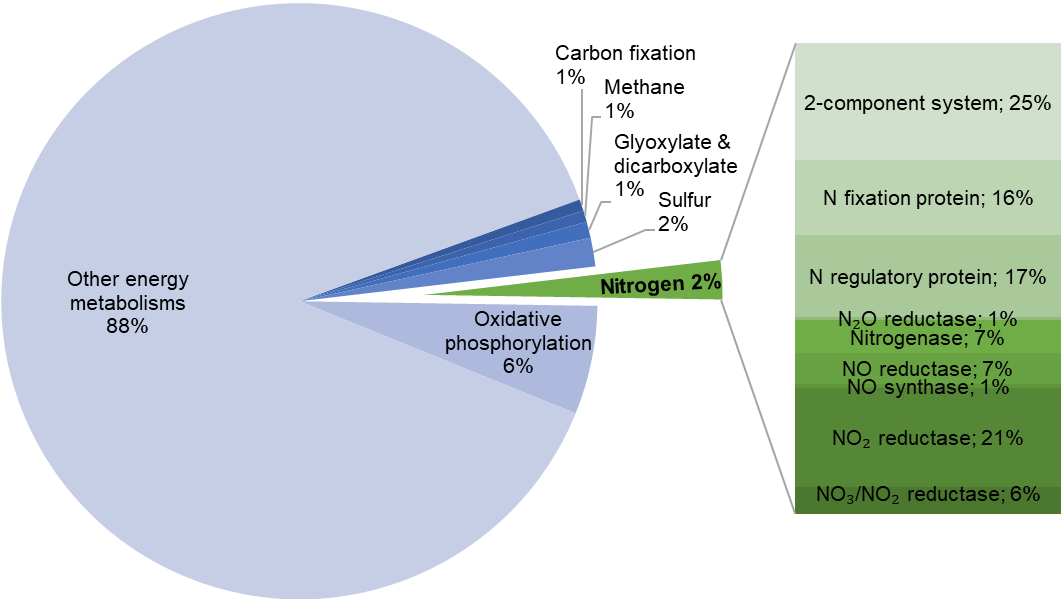


Figure S9. Bacterial energy metabolism.


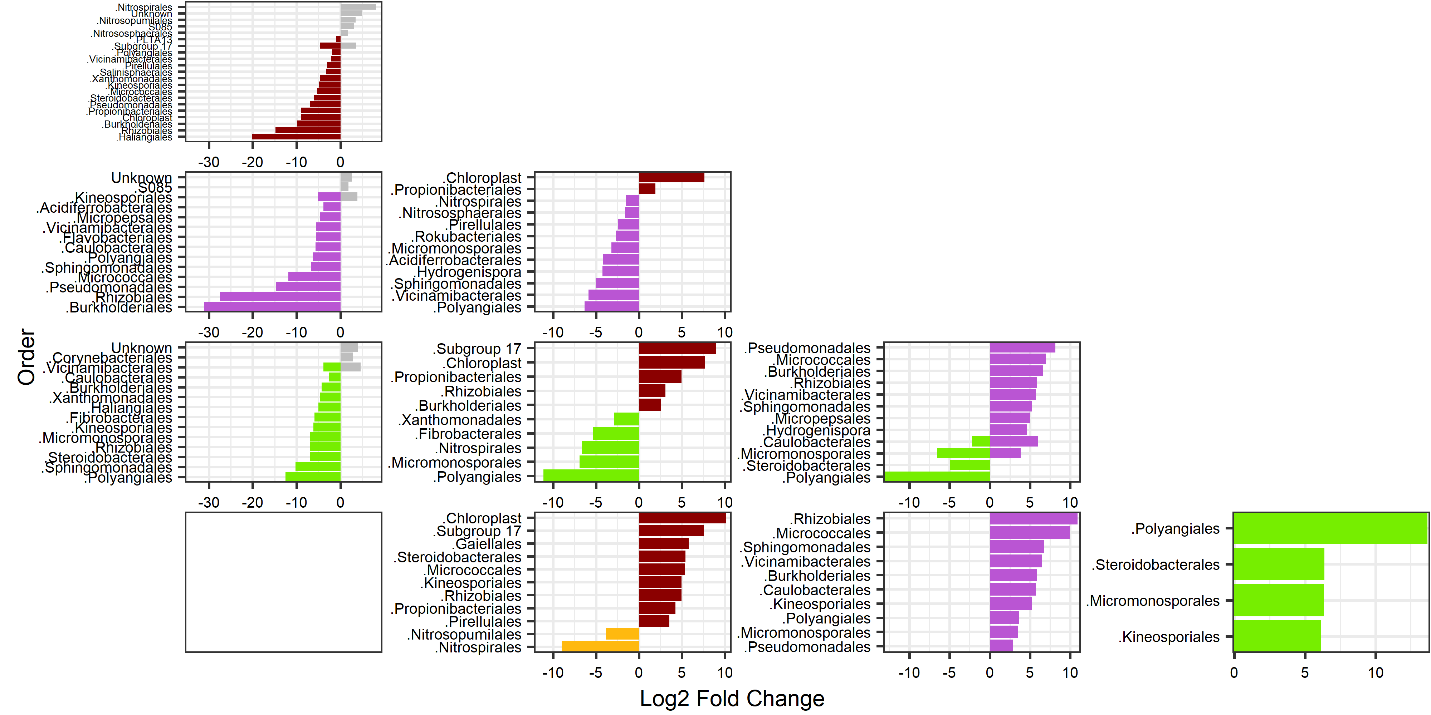


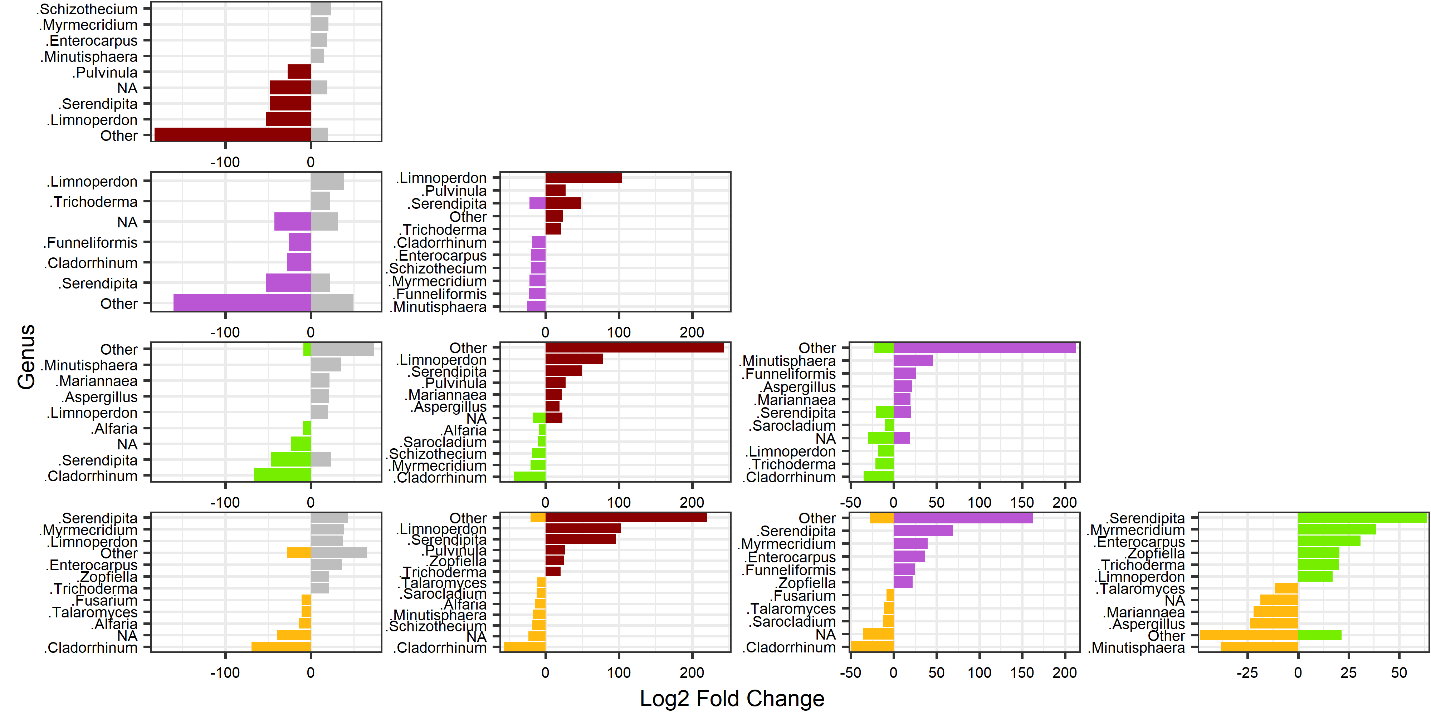

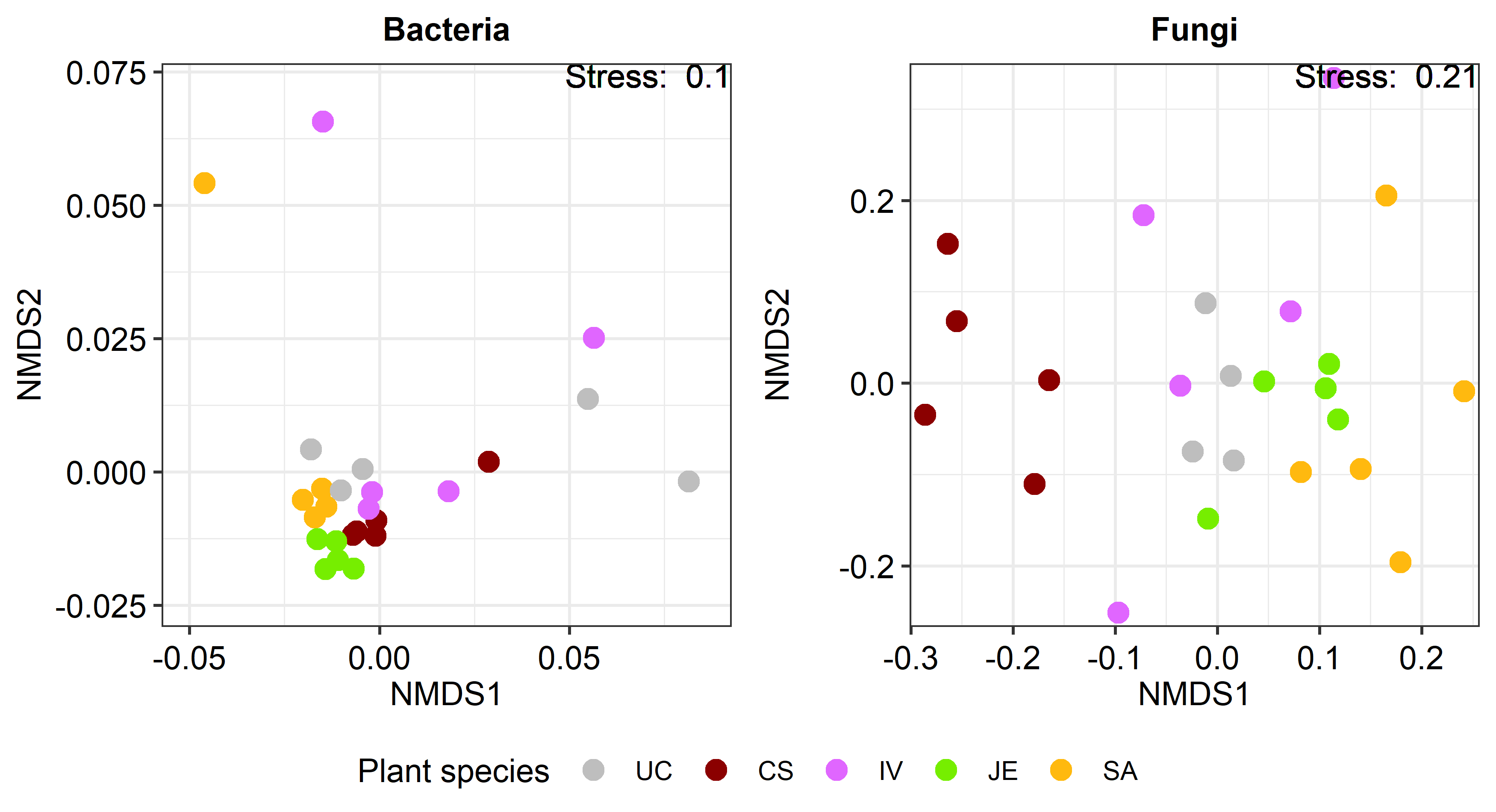


Figure S10. 2 to 2 comparisons between planted species, of significantly different bacterial Order or fungal Genus’s abundances, before saline runoff; CS: *Cornus sericea*, JE: *Juncus effusus*, IV: *Iris versicolor*, SA: *Sesleria autumnalis*, UC: unplanted.

**A**
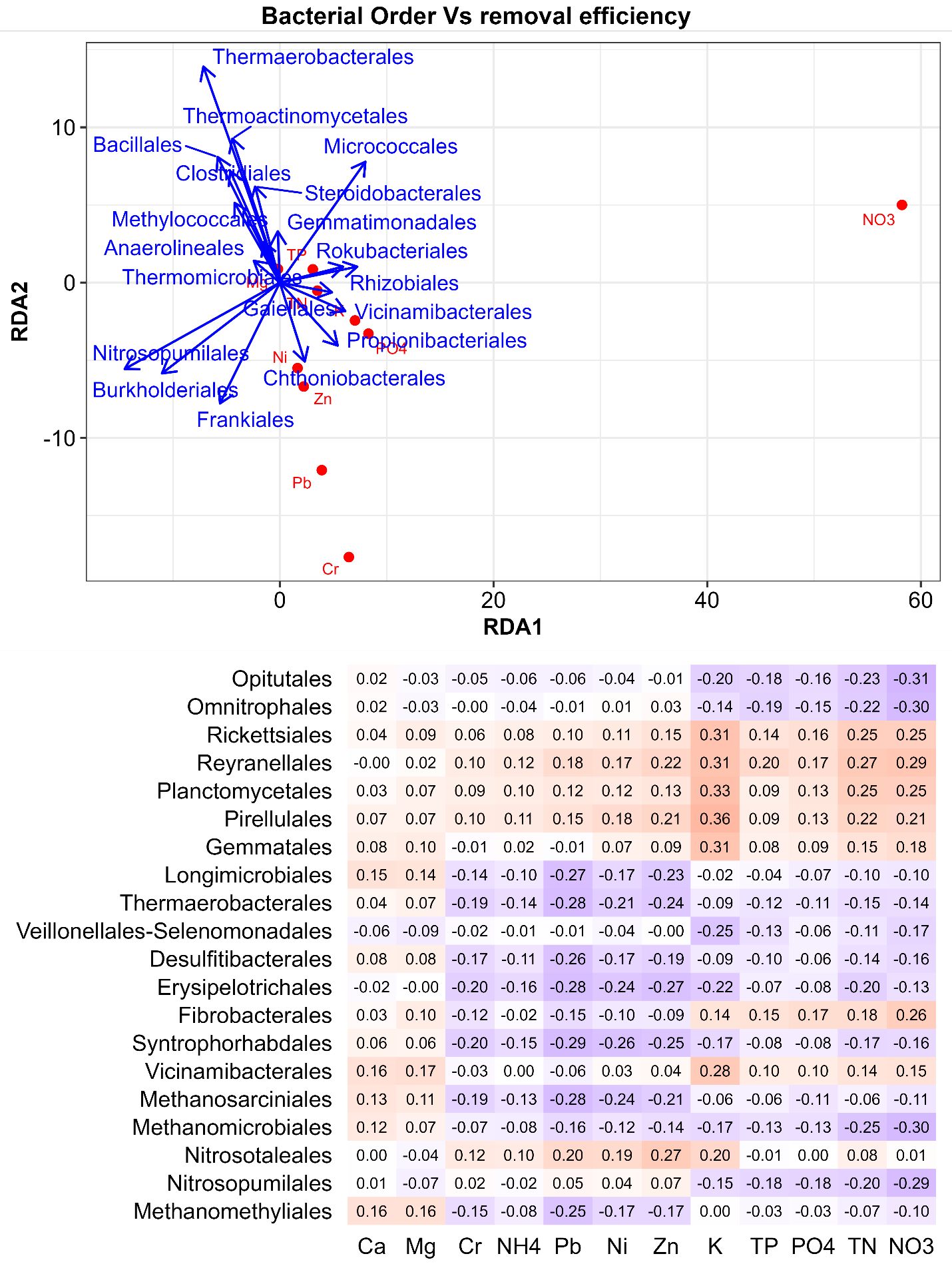


**B**
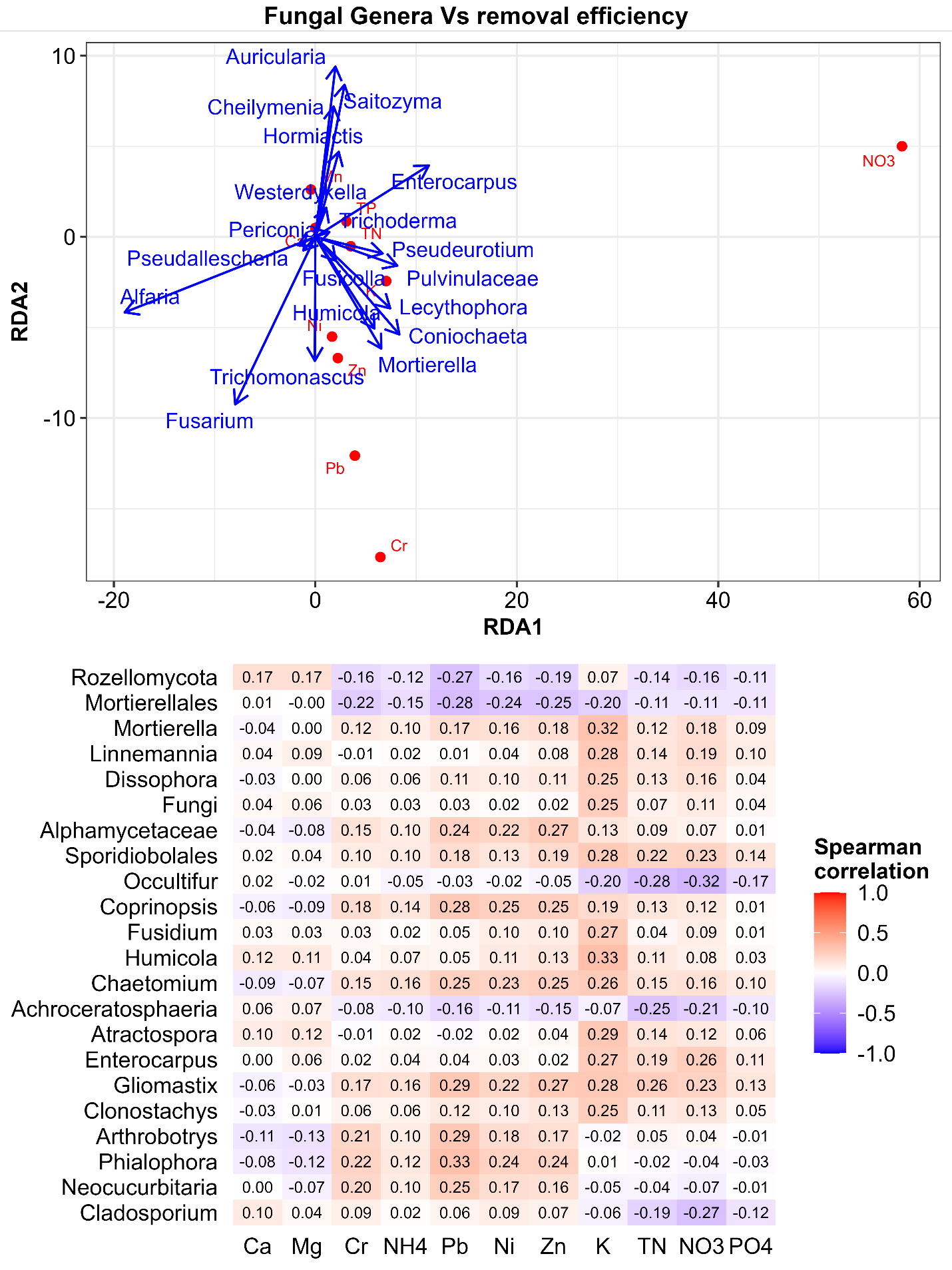


Figure S11. Redundancy Analysis (RDA) and table of top bacterial (A) and fungal (B) Spearman correlations with BR removal performance efficiency.
